# A Review of Benzophenone-Based Derivatives for Organic Light-Emitting Diodes

**DOI:** 10.3390/nano14040356

**Published:** 2024-02-14

**Authors:** Dovydas Blazevicius, Saulius Grigalevicius

**Affiliations:** Department of Polymer Chemistry and Technology, Kaunas University of Technology, Radvilenu Plentas 19, LT50254 Kaunas, Lithuania

**Keywords:** donor–acceptor–donor (D–A–D) derivatives, organic light-emitting diode (OLED), high efficiency, emission, thermal analysis, synthesis

## Abstract

Organic light-emitting diodes (OLEDs) have garnered considerable attention in academic and industrial circles due to their potential applications in flat-panel displays and solid-state lighting technologies, leveraging the advantages offered by organic electroactive derivatives over their inorganic counterparts. The thin and flexible design of OLEDs enables the development of innovative lighting solutions, facilitating the creation of customizable and contoured lighting panels. Among the diverse electroactive components employed in the molecular design of OLED materials, the benzophenone core has attracted much attention as a fragment for the synthesis of organic semiconductors. On the other hand, benzophenone also functions as a classical phosphor with high intersystem crossing efficiency. This characteristic makes it a compelling candidate for effective reverse intersystem crossing, with potential in leading to the development of thermally activated delayed fluorescent (TADF) emitters. These emitting materials witnessed a pronounced interest in recent years due to their incorporation in metal-free electroactive frameworks and the capability to convert triplet excitons into emissive singlet excitons through reverse intersystem crossing (RISC), consequently achieving exceptionally high external quantum efficiencies (EQEs). This review article comprehensively overviews the synthetic pathways, thermal characteristics, electrochemical behaviour, and photophysical properties of derivatives based on benzophenone. Furthermore, we explore their applications in OLED devices, both as host materials and emitters, shedding light on the promising opportunities that benzophenone-based compounds present in advancing OLED technology.

## 1. Introduction

Since C. W. Tang’s groundbreaking research in 1986 [1], there has been significant interest in organic light-emitting diodes (OLEDs) for several decades. Their potential applications span a wide range including full-colour flat-panel displays, smart watches, smartphones, large-screen televisions, and solid-state lighting, attracting attention in both scientific and industrial domains [2,3,4,5,6,7,8]. Today, OLEDs have evolved into a crucial commercial venture, driven by their notable advantages in terms of brightness, wide-angle viewability, power efficiency, contrast ratio, and other factors [9,10,11,12,13,14,15,16,17,18,19,20]. Flexibility stands out as an additional attribute in their technological arsenal, unmatched by any of the other existing technologies [21,22]. Predictably, the relentless pursuit of superior OLED materials continues, driven by the quest for enhanced efficiency when incorporated into devices.

**Figure 1:**
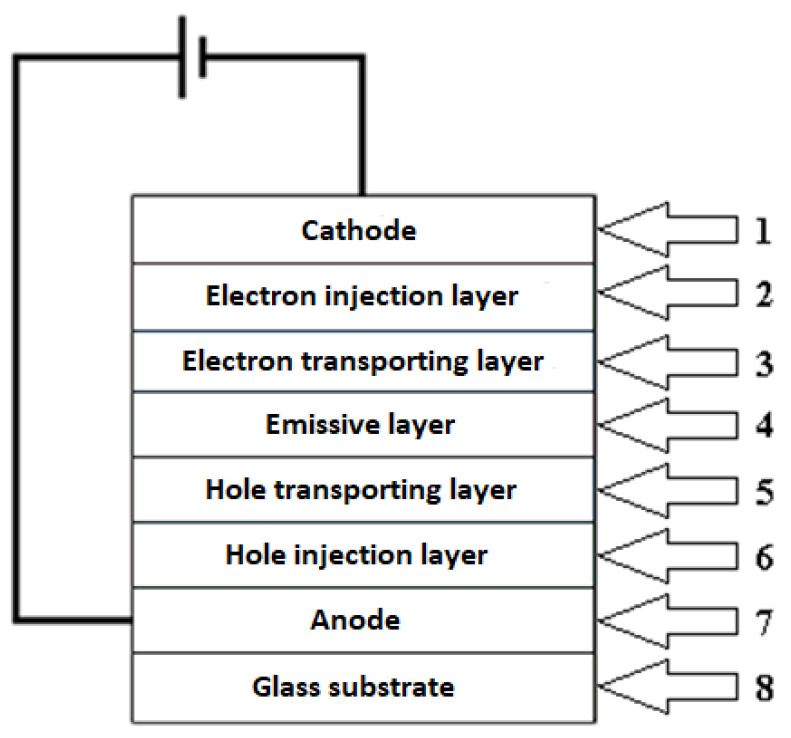
Structure of organic light-emitting diode.

Originally, OLEDs were simple devices with a single emissive layer placed between a cathode and an anode, deposited on a transparent substrate-like glass. As interest in the technology grew, and the pursuit of higher efficiency intensified, additional layers were introduced to optimize the flow of electrons and holes to the emissive layer. This is where excitons form, leading to photon generation during the recombination process. In Figure 1, we illustrate a modern multi-layer OLED structure. Beginning with a transparent substrate, an anode is deposited, typically made of the low-resistance transparent material indium tin oxide (ITO). Subsequently, hole injection (HIL) and hole transporting (HTL) layers are added. For these layers, materials with high hole mobility and robust electron-donating properties are chosen. Following that, the emissive layer (EML) is introduced, composed either of the emitting material alone or doped within a host matrix. Materials in this layer commonly feature both electron-donating and electron-accepting groups, essential for an efficient recombination process. The primary purpose of the host material is to effectively transfer charge to the emitter while avoiding various emission-quenching mechanisms. OLED emitters are categorized into three generations: 1st-generation fluorescent, 2nd-generation phosphorescent, and 3rd-generation thermally activated delayed fluorescence (TADF) phenomena featuring emitters. Moving forward, electron transporting (ETL) and electron injection (EIL) layers are applied, performing the opposite functions to HTL and HIL. These layers utilize materials with high electron mobility and robust electron-accepting properties. Finally, a cathode, often made of aluminium, is deposited. Presently, there is a diverse range of OLED structures, some incorporating even more layers (electron/hole blocking, multiple charge-transporting layers, etc.) than the example provided. This complexity aims to achieve well-aligned energy levels for each layer with its adjacent layers, thereby minimizing energy barriers for charge carrier transport and achieving more efficient OLED devices.

In past decades, there has been a progressive transition from fluorescence- to phosphorescence-based devices in the pursuit of higher efficiencies [23,24,25,26,27]. OLEDs utilizing conventional fluorescent emitters typically exhibit a maximum internal quantum efficiency (IQE) of 25% [1]. IQE can be elevated from 25% to 100% by harnessing triplet excitons through the use of intersystem singlet-to-triplet crossing in phosphorescent emitters [28,29]. Despite the high internal quantum efficiency and operational stability of emissive iridium or platinum complexes [30,31,32,33], phosphorescent OLEDs (PhOLEDs) encounter significant efficiency roll-off due to issues like aggregation-caused quenching and triplet–triplet and triplet–polaron annihilation [34,35,36,37]. As a result, host–guest systems are commonly employed to disperse phosphorescent emitters into host matrices. To achieve high-performance PhOLEDs, host materials must be ingeniously designed to adhere to fundamental principles such as good thermal stability and film formation quality [38,39,40,41,42,43] and a sufficiently high triplet energy to prevent reverse energy transfer from emitter to host [44,45,46]. Nevertheless, the persistent issue of poor stability of blue phosphorescent OLEDs remains a significant constraint [47,48]. The presence of noble metals in phosphorescent materials raises an undeniable challenge to the future costs of devices and environmental considerations [49,50]. In recent years, there has been a notable focus on TADF emitters. This interest arises from their utilization in metal-free electroactive frameworks and their ability to upconvert full triplet excitons into emissive singlet excitons through reverse intersystem crossing (RISC), thereby achieving ultrahigh external quantum efficiencies (EQEs) [51,52,53,54,55,56,57]. Despite the promise of TADF materials, many TADF OLEDs encounter same quenching and triplet–triplet (or singlet–triplet) annihilation problems as PhOLEDs, due to a long exciton lifetime [58,59,60]. Consequently, to address the concentration quenching problem, many TADF molecules also need to be incorporated into suitable host matrices. However, because of a limited selection of TADF-specific host materials [61,62,63], researchers often utilize conventional host materials common to phosphorescent OLEDs [64]. Therefore, one approach to overcoming efficiency challenges is the design of new host materials specifically tailored for TADF OLEDs. Another approach involves the development of new TADF molecules, especially for non-doped OLEDs based on materials exhibiting aggregation-induced emission (AIE) characteristics [65,66,67,68,69,70,71].

**Figure 2:**
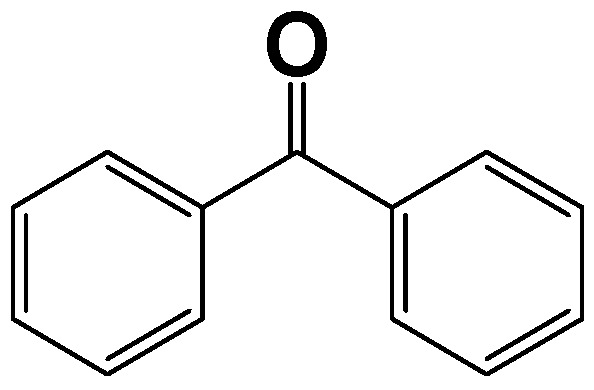
Chemical structure of benzophenone.

Units derived from benzophenone, shown in Figure 2, are renowned for their efficient intersystem crossing (ISC) capability, attributed to robust spin–orbit coupling [72]. This characteristic renders them highly appealing as acceptor blocks for the fabrication of TADF emitters. Notably, benzophenone is recognized for its stability as an acceptor [73]. Furthermore, benzophenone functions as a classical phosphor with a high intersystem crossing efficiency, potentially leading to effective reverse intersystem crossing with a small ΔE_ST_ [74]. The benzophenone framework not only serves as an electron-deficient core by integrating various donor units to create molecules with small ΔE_ST_ and intramolecular charge transfer (CT) states [75], but it also features a highly twisted geometry, reducing intermolecular interactions and the self-quenching effect [76,77]. Owing to the properties of benzophenone, most of its derivatives have found applications in emissive layers of OLEDs both as a host or as an emitter.

In this review article, we examine the synthetic pathways, thermal characteristics, electrochemical behaviour, photoelectrical, and photophysical properties of derivatives based on benzophenone. Additionally, we explore their applications in OLED devices, both as host materials as well as emitters. This article will systematically review various device structures and their corresponding performances. This review is structured into several sections based on the application and structure of benzophenone derivatives, including the following: host materials for phosphorescent emitters, host materials for TADF emitters, donor–acceptor (D–A)-type emitters, donor–acceptor–donor (D–A–D)-type symmetric structure emitters, D–A–D-type asymmetric structure emitters, as well as emitters having dendritic structures.

## 2. Benzophenone-Based Host Materials Used for Phosphorescent Emitters

**Scheme 1:**
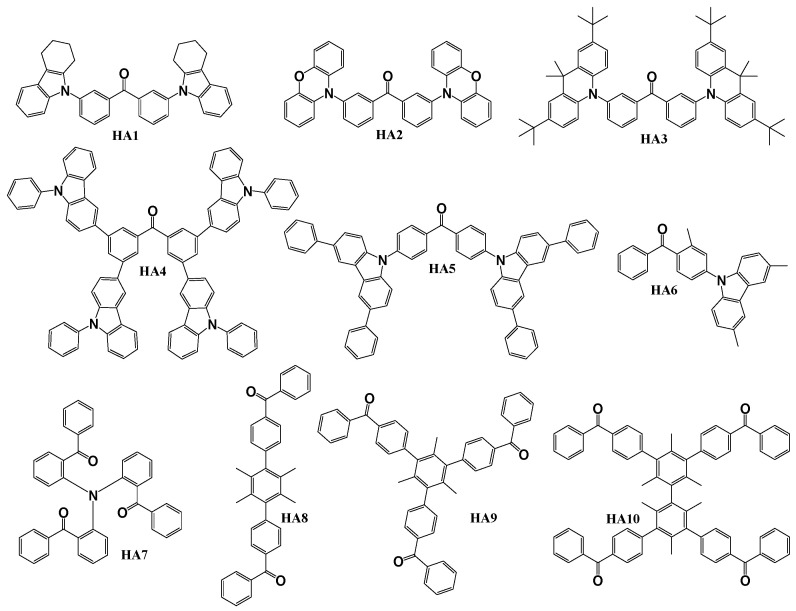
Structures of benzophenone-based materials used as hosts in PhOLEDs.

Scheme 1 illustrates the configurations of benzophenone-based derivatives, employed as host materials in PhOLED devices. The objective compounds HA1, HA2, and HA3 [78] were obtained using Buchwald–Hartwig [79] amination reaction between 3,3′-dibromobenzophenone and 1,2,3,4-tetrahydrocarbazole for HA1, phenoxazine for HA2, and 9,9-dimethyl-9,10-dihydroacridine for HA3. The host material HA4 [80] was synthesized by utilizing Suzuki coupling reaction [81] between 3,3′,6,6′-tetrabromobenzophenone and 9-phenyl-9H-carbazol-3-ylboronic acid. To obtain the target compound HA5 [82], Buchwald–Hartwig amination reaction of 4,4′-dibromobenzophenone with a 3,6-diphenyl-9H-carbazole donor unit was used. Material HA6 [83] was synthesized utilizing similar reaction conditions between 4-bromo-3-methylbenzophenone and 3,6-dimethyl-9H-carbazole. The host material HA7 [84] was acquired through a single-step synthetic procedure employing Ullmann reaction [85] between iodophenylketone and aminodiphenylketone. The synthesis of the remaining PhOLED host materials HA8, HA9, and HA10 [86] was easily achieved through a simple Friedel–Crafts benzoylation reaction [87] carried out in carbon disulfide. Benzoyl chloride, in the presence of AlCl_3_, reacted with 1,4-diphenyldurene, 1,3,5-triphenylmesitylene, and 3,3′,5,5′-tetraphenylbimesitylene to yield HA8, HA9, and HA10, respectively.

Thermal, electrochemical, photoelectrical, and photophysical properties of the materials HA1–HA10 are displayed in Table 1. The thermal properties were investigated for all the objective materials. All the derivatives showed good thermal stability with high thermal decomposition temperatures (T_D_) of 218–553 °C, as confirmed by thermogravimetric analysis (TGA). Differential scanning calorimetry (DSC) measurements showed that the derivatives HA1, HA4, HA6, HA7, and HA10 were fully amorphous materials with glass transition temperatures (T_G_) of 55–188 °C. Only melting temperatures (T_M_) of 118–370 °C were detected for other presented materials. During cooling, crystallization temperatures (T_Cr_) of 62 °C and 269 °C were detected for compounds HA2 and HA3, respectively. For this group of materials, HOMO levels were measured to be from −5.80 eV to −4.74 eV and LUMO levels were in a region of −2.80 eV to −1.83 eV, attributed to bandgap energies (E_g_) of 2.72–3.93 eV. Benzophenone-based PhOLED host materials showed high triplet state energy (E_T_) levels, which were in a range of 2.53 eV–3.02 eV. Regarding photoluminescence quantum yield, materials with this metric measured demonstrated 15–24.4% quantum yield from film (Φ_PL_ film) and 3–15.3% quantum yield from solution (Φ_PL_ sol). In terms of photoluminescence quantum yield, materials with this metric measured showed a range of 15% to 24.4% in film (Φ_PL_ film) and 3% to 15.3% in solution (Φ_PL_ sol). All these properties were suitable to test the materials as hosts for various phosphorescent emitters. 

Architectures of devices utilizing host materials HA1–HA10 are shown in Table 2. While all the devices used indium tin oxide (ITO) as the anode, hole-transporting layers (HTLs) had more variability. All the reviewed devices used two HTLs of different derivatives stacked on top of each other. Various materials were used in forming HTLs, such as 1,1-bis[(di-4-tolylamino)phenyl]cyclohexane (TAPC), 1,3-bis(N-carbazolyl)benzene (mCP), poly(3,4-ethylenedioxythiophene):polystyrene sulfonate (PEDOT:PSS), N,N′-di(1-naphthyl)-N,N′-diphenyl-(1,1′-biphenyl)-4,4′-diamine (NPB), tris(4-carbazoyl-9-ylphenyl)amine (TCTA), and 1,4,5,8,9,11-hexaazatriphenylenehexacarbonitrile (HAT-CN). In emissive layers, iridium-based phosphorescent emitters were doped in host materials HA1–HA10, using various doping concentrations. Bis(1-phenylisoquinoline)(acetylacetonate)iridium(III) (Ir(piq)2acac), bis[2-(3,5-dimethylphenyl)-4-methyl-quinoline](acetylacetonate)iridium(III) (Ir(mphmq)2(tmd)), bis(2-methyldibenzo[f,h]quinoxaline)(acetylacetonate)iridium(III) (Ir(MDQ)2acac), bis(2-benzo[b]thiophen-2-yl-pyridine)(acetylacetonate)iridium(III) (Ir(btp)2acac), and bis(2-benzothiozolato-phenyl)(pylbenzamidinate)iridium(III) (Ir(bt)2(dipba)) were used for red, bis(7,8-benzoquinolinato)(N,N′-diisopropylbenzamidine) iridium (III) (Ir(bzq)2(dipba)) for orange, bis(4-phenylthieno[3,2-c]pyridinato-N,C2′) (acetylacetonate) iridium(III) (PO-01) for yellow, bis[2-(2-pyridinyl-N)phenyl-C](acetylacetonato)iridium(III) (Ir(ppy)2acac) and tris(2-phenylpyridine)iridium(III) (Ir(ppy)3) for green, and bis[2-(4,6-difluorophenyl)pyridinato-C2,N](picolinato)iridium (FIrpic) for blue devices. For electron-transporting layers (ETLs), 4,6-bis(3,5-di(pyridin-3-yl)phenyl)-2-methylpyrimidine (B3PYMPM), 4,6-bis(3-(9H-carbazol-9-yl)phenyl)pyrimidine (CzPhPy), and 1,3,5-tris(3-pyridyl-3-phenyl)benzene (TmPyPB) were used. Additionally, 4,7-diphenyl-1,10-phenanthroline (Bphen), lithium fluoride (LiF), lithium-8-hydroxyquinolinolate (Liq), or caesium carbonate (Cs_2_CO_3_) were deposited as electron-injecting layers (EILs). Lastly, all of the devices mentioned in this section used aluminium (Al) as a cathode.

Characteristics of phosphorescent OLED devices using host materials HA1–HA10 are shown in Table 3. Overall, the utilization of benzophenone-based host materials resulted in efficient PhOLED devices with the external quantum efficiency of most devices exceeding 10%. Host materials HA1–HA10 were used for red, orange, yellow, green, and blue devices. Comparison of the red devices with each other showed that the most efficient prototype was D3HA5, which has a demonstrated low turn-on voltage (V_ON_) of 2.9 V, high power efficiency (PE) of 27.1 lm/W, and external quantum efficiency (EQE) of 22.1%, as well as a maximum brightness (L_MAX_) of 10,240 cd/m^2^. For the orange emitter, only 3,6-diphenylcarbazol-9-yl-substituted host HA5 was used. The device D2HA5 demonstrated a V_ON_, L_MAX_, PE, and EQE of 2.6 V, 31,200 cd/m^2^, 61.6 lm/W, and 23.1%, respectively, with CIExy coordinates of (0.51, 0.47). Between yellow devices, prototype D4HA8 stood out the most and outperformed devices D4HA9 and D4HA10. That could be attributed to the structure of host material HA8 which utilizes two benzophenone fragments compared to the three of HA9 and four of HA10. Diode D4HA8 demonstrated a current efficiency (CE) of 50.6 cd/A, PE of 28.9 lm/W, and EQE of 19.2%. Comparing green PhOLEDs, two carbazole fragments having the host material HA5 outperformed other host materials that had one or four carbazole fragments (HA4, HA6) or without carbazole fragments (HA8, HA9, HA10). Furthermore, the suitability of derivative HA5 (E_T_ = 2.69 eV) for green phosphors could be ascribed to the highest efficiency observed in the devices. PhOLEDs with a D1HA5 host demonstrated a very high PE of 99.1 lm/W and excellent EQE of 25.1% with their L_MAX_ exceeding 93,300 cd/m^2^. When comparing blue PhOLEDs, devices using host material HA6 with a high E_T_ of 3.00 eV and excellent thermal and film forming properties exhibited the best characteristics. Device D1HA6 displayed a high PE and EQE of 38.2 lm/W and 19.4%, respectively.

## 3. Benzophenone-Based Bipolar Host Materials Used for TADF Emitters

**Scheme 2:**
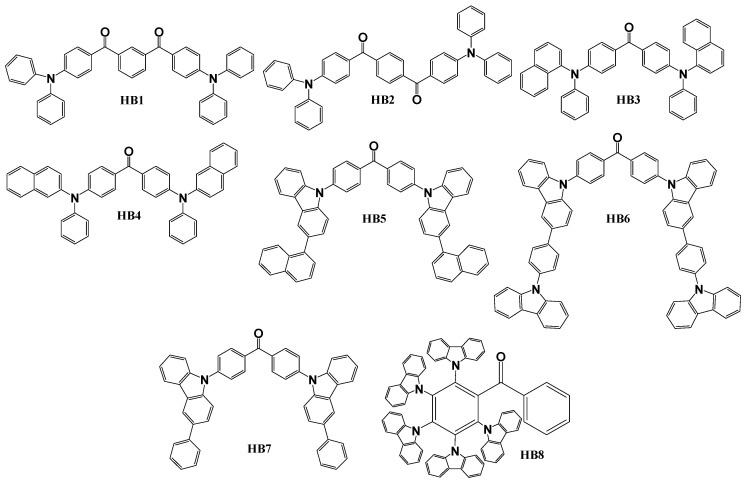
Structures of benzophenone-based materials used as hosts in TADF OLEDs.

Scheme 2 illustrates the configurations of benzophenone-based derivatives, employed as host materials in TADF OLED devices. Objective compounds HB1 and HB2 [88] were synthesized via a straightforward one-step Friedel–Crafts reaction utilizing readily available, cost-effective triphenylamine and isophthaloyl or terephthaloyl dichloride as initial reagents. All the remaining host materials HB3, HB4 [89], HB5 [90], HB6, HB7 [91], and HB8 [92] were obtained by simple nucleophilic substitution reactions between fluorinated benzophenone and corresponding amines. For application as TADF host materials, benzophenone was combined either with carbazole or phenyl-/naphtyl- amino fragments as electron donors. 4,4′-Difluorobenzophenone reactions with N-phenyl-1-naphtylamine, N-phenyl-2-naphtylamine, 3-naphthyl-9H-carbazole, 3-phenyl-9H-carbazole, and 3-(4-(9H-carbazol-9-yl)phenyl)-9H-carbazole yielded compounds HB3, HB4, HB5, HB6, and HB7, respectively. Material HB8 was obtained during reaction between 2,3,4,5,6-pentafluorobenzophenone and 9H-carbazole.

Table 4 presents the thermal, electrochemical, photoelectrical, and photophysical properties of materials HB1 to HB8. During the investigation of thermal properties, it was noticed that most of the benzophenone-based compounds were resistant to heat with thermal decomposition temperatures ranging from 277 °C to as high as 497 °C. Most of the same materials also possessed the ability to form stable amorphous films with glass transition temperatures ranging from 90 °C to 187 °C. At the same time, HB series benzophenone derivatives had optical bandgap energies of 2.70–4.10 eV, singlet state energy (E_S_) levels of 2.37–3.07 eV, and triplet state energies ranging from 2.32 eV to 2.64 eV. Notably, carbazole-substituted derivatives HB5-HB8 demonstrated higher E_T_ levels.

The configurations of devices employing host materials HB1 to HB8 are illustrated in Table 5. All the manufactured devices used an ITO anode. To lower the hole injection barrier, molybdenum (VI) oxide (MoO_3_) was used as a hole injection layer (HIL) for devices DHB3 and DHB4. The most popular material of choice for the formation of hole-transporting layers was PEDOT:PSS. NPB, TAPC, or a cross-linkable molecule 3,6-bis(4-vinylphenyl)-9-ethylcarbazole (VPEC), were also employed for HTLs in some cases. Each device utilized one or two hole-transporting layers (HTLs). The chosen TADF emitters included orange 1,2,3,5-tetrakis(carbazol-9-yl)-4,6-dicyanobenzene (4CzTPN), green 2,3,5,6-tetracarbazole-4-cyano-pyridine (4CzCNPy), and blue 10,10′-(perfluoro-[1,10-biphenyl]-4,4′-diyl)bis(2,7-ditert-butyl-9,9-dimethyl-9,10-dihydro-acridine) (PFBP-2b). The electron-transporting layers utilized compounds such as TmPyPB, TPBi, and 2,4,6-tris[3-(diphenylphosphinyl)phenyl]-1,3,5-triazine (PO-T2T). Finally, for all devices under investigation, a combination of LiF as the electron-injecting layer and an Al cathode was employed.

Measured characteristics of TADF OLED devices employing host materials HB1–HB8 are shown in Table 6. Most of the HB materials were used to dope green TADF emitters, except benzophenone derivatives HB3 and HB5, which were used in combination with orange and sky blue dopants to achieve white emission. Hu and co-authors investigated electron-donating triphenylamino and electron-accepting phthaloyl moieties having compounds HB1 and HB2. When these materials were used as hosts for the green TADF emitter, compound HB1 prevailed as a more effective choice that could be explained by higher singlet and triplet energy levels which enhance energy transfer efficiency and effectively prevent reverse energy transfer from the green TADF emitter to the TADF host, resulting in a CE, PE, and EQE of 43.5 cd/A, 33.3 lm/W, and 13.0% for device DHB1, respectively. Materials HB3 and HB4 were investigated by Mahmoudi et al. They found that the best use of the synthesized materials would be as exciton modulators between two TADF emitters. By combining orange and blue TADF materials with the new benzophenone-based hosts, efficient white TADF OLEDs were introduced with the EQE for devices DHB3 and DHB4 reaching 9.5% and 7.1%, respectively. Device-utilizing compound HB3 showed higher-quality white electroluminescence, which is defined by CIE coordinates of (0.32, 0.31), a colour temperature of 4490 K, and a colour-rendering index of 80. A team of researchers led by Swyamprabha synthesized and characterized 3-naphtyl-9H-carbazole-substituted derivative HB5. The green TADF OLED device showed a maximum CE, PE, and EQE of 9.5 cd/A, 8.4 lm/W and 2.8%, respectively. The same researchers continued their work and synthesized new materials HB6 and HB7 with different substituents at the carbazole core. At last, green solution-processable OLEDs were also fabricated with a cross-linkable hole transport material VPEC and realized PE of 63.6 lm/W with an EQE of 25.3% for device D2HB7, which was more effective than an analogical device with a lower E_T_ having host material HB6. Wang et al. introduced a new penta-carbazole-substituted benzophenone derivative HB8, which has also been tested as a host material for the green TADF emitter. Device DHB8 displayed a yellowish-green emission, possessing CIE coordinates of (0.34, 0.58), aligning with the emission characteristics of 4CzCNPy [93]. The OLED achieved a maximum current and external quantum efficiency, reaching 38.3 cd/A and 12.5%, respectively.

## 4. Benzophenone-Based Emitters Employing a D–A Molecular Structure

**Scheme 3:**
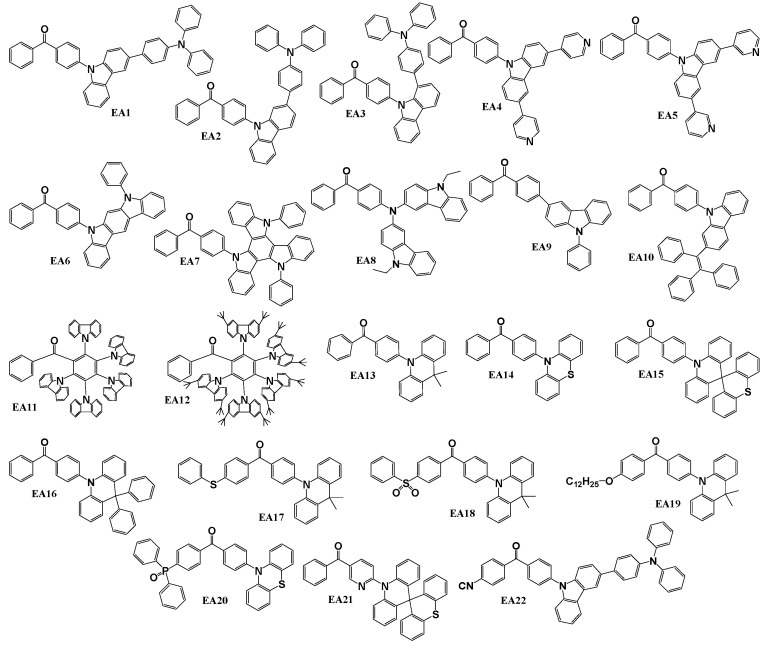
Structures of benzophenone-based D–A materials used as emitters in OLEDs.

Scheme 3 illustrates the structures of donor–acceptor-type molecules reviewed for their application as emitters in OLED devices. These materials typically exhibit a structure where one part of the molecule has a higher electron-donating ability, while another part has a higher electron-accepting ability. The compounds EA1, EA2, EA3 [94], EA6 [95], EA7 [96], EA8 [97], EA13 [98], EA14 [99], EA16 [100], EA17, and EA18 [101] were synthesized using the Buchwald–Hartwig coupling reaction methodology. Specifically, EA1, EA2, and EA3 were obtained in the reaction of 4-bromobenzophenone with 3-(4-(diphenylamino)phenyl)-9H-carbazole, 2-(4-(diphenylamino)phenyl)-9H-carbazole, and 1-(4-(diphenylamino)phenyl)-9H-carbazole, respectively. EA6 and EA7 were derived from the reactions of 4-bromobenzophenone with 11-phenyldihydroindolo[2,3-a]carbazole or N,N′-diphenyltriazatruxene. EA8 resulted from the reaction of 4-aminobenzophenone with 3-iodo-9-ethylcarbazole. Emitters EA13, EA14, and EA16 were synthesized through the reactions of 4-bromobenzophenone with 9,9-dimethyl-9,10-dihydroacridine, 10H-phenothiazine, or 9,9-diphenyl-9,10-dihydroacridine, respectively. Lastly, employing 9,9-dimethyl-9,10-dihydroacridine in reaction with (4-bromophenyl)(4-(phenylthio)phenyl)methanone resulted in EA17 and 9,9-dimethyl-9,10-dihydroacridine reaction with (4-bromophenyl)(4-(phenylsulfonyl)phenyl)methanone, which was used to obtain EA18. Materials EA4 and EA5 [102], featuring 3,6-pyridinyl-9H-carbazole substitutions, were synthesized through Suzuki reactions. This involved the coupling of 4-(3,6-dibromocarbazol-yl)benzophenone with 4-pyridinylboronic acid for the production of compound EA4 or with 3-pyridinylboronic acid for the synthesis of derivative EA5. The Suzuki coupling method was also employed for the synthesis of target materials EA9 [103] and EA22 [104]. For EA9, the reaction involved 4-bromobenzophenone with 9-phenylcarbazole-3-boronic acid pinacol ester. For derivative EA22, the initial reagents selected were 4-(4-(3-bromo-9H-carbazol-9-ylbenzoyl)benzonitrile and (4-(diphenylamino)phenyl)boronic acid. Benzophenone-based derivatives EA10 [105], EA11 [92], EA12 [106], and EA19 [107] were synthesized through relatively straightforward catalyst-free nucleophilic substitution reactions. For EA10, the initial reactants included 4-fluorobenzophenone and triphenyl-2-(9H-carbazol-2-yl)ethylene. The synthesis of material EA11 is detailed in a previous section under the name HB8. The objective compound EA12 was obtained by reacting of 2,3,4,5,6-pentafluorobenzophenone with 3,6-bis(tert-butyl)-9H-carbazole. Emitter EA19 was prepared through the reaction of (4-(dodecyloxy)phenyl)(4′-fluorophenyl)methanone with 9,9-dimethyl-9,10-dihydroacridine. An alternative synthesis approach was employed for the creation of compounds EA15 and EA21 [108]. In this scenario, 10H-spiro(acridine-9,9′-thioxanthene) was employed in Ullmann reactions with 4-bromobenzophenone and (6-bromopyridin-3-yl)(phenyl)methanone, respectively. The phosphorus-containing emitter EA20 [109] was synthesized through a palladium-catalysed reaction between the intermediate compound 4-iodo-4′-phenothiazin-10yl-benzophenone and diphenylphosphine oxide in the presence of triethylamine.

The thermal, electrochemical, photoelectrical, and photophysical properties of materials EA1–EA22 are provided in Table 7. All the tested materials exhibited good thermal stability, as verified by the TGA measurements conducted on the samples. The thermal decomposition temperatures for these materials ranged from 278 °C to 497 °C. DSC experiments showed that most of the tested materials were capable of forming stable amorphous layers with glass transition temperatures of 80–194 °C. By analysing the HOMO–LUMO gap (energy bandgap E_g_), it was seen that most of the EA series derivatives demonstrated E_g_ levels of around 3 eV or slightly lower. Only materials EA4 and EA5 showed significantly higher E_g_ levels of around 4 eV, which might lead to less efficient TADF processes compared to emitters with smaller bandgaps [59]. A crucial characteristic for achievement of the TADF effect is a small energy difference between the singlet and triplet states (ΔE_ST_), which facilitates effective reverse intersystem crossing, allowing the conversion of triplet excitons to singlet excitons, which is essential for delayed fluorescence [59]. This metric was measured for all benzophenone-based D–A emitters. From 22 derivatives described in this section, 17 of them demonstrated ΔE_ST_ levels of 0.10 eV or lower. Higher singlet–triplet energy difference-having derivatives could be suffering from an ineffective TADF process, thus lowering the overall performance of the devices. A small ΔE_ST_ is associated with a higher photoluminescence quantum yield (Φ_PL_), as it facilitates efficient radiative decay from the triplet state to the ground state. We can compare materials EA1, EA2, and EA3 since experiments with them were executed under the same conditions. The biggest ΔE_ST_ of 0.37 eV having derivative EA2 showed the lowest quantum yield (Φ_PL_) of 20% in thin film. On the other hand, the narrowest singlet–triplet energy gap of just 0.02 eV was measured for compound EA3, which exhibited the highest Φ_PL_ of 50%. This trend could be observed in other pairings of similarly structured materials that were characterized under the same conditions such as EA4 and EA5 as well as EA15 and EA21. Oxygen molecules can quench the triplet states involved in the TADF process [110]. This quenching effect can lead to a decrease in the efficiency of delayed fluorescence and, consequently, reduced performance of TADF-based devices. Triplet state involvement in overall emission is proven by this way for benzophenone derivatives EA5, EA6, EA8, and EA11. In the cases of materials EA6, EA13, EA15, EA16, and EA21, prompt and delayed components of emissions were detected with the ratio of the delayed component in overall emission (R_D_) ranging from 37.9% to 81.3%. All emitters described in this section, except fluorescent EA10, exhibited TADF properties.

Architectures of devices utilizing emitters EA1–EA22 are displayed in Table 8. As it was mentioned in earlier sections, all of the devices formed with the D–A-type benzophenone emitters used an ITO anode and Al cathode except for EA20-based devices, which used cathodes made of a blend of magnesium and silver. To lower the hole injection barrier, hole-injecting material MoO_3_ was used in some devices. Hole-transporting layers were made utilizing PEDOT:PSS, NPB, TAPC, HAT-CN, TCTA, 9-(4-tert-butylphenyl)-3,6-bis(triphenylsilyl)-9H-carbazole (CzSi), mCP, 3,3′-di(9H-carbazol-9-yl)-1,1′-biphenyl (mCBP), 4,4′-bis(9-carbazolyl)-1,1′-biphenyl (CBP), or N,N′-bis(naphthalen-1-yl)-N,N′-bis(phenyl)-2,2′-dimethylbenzidine (α-NPD). Doped and non-doped emissive layers (EMLs) were used during studies of the devices. In the case of doped devices, host materials bis[2-(diphenylphosphino)phenyl]ether oxide (DPEPO), 3,6-di(9-carbazolyl)-9-(2-ethylhexyl)carbazole (TCzl), mCP, bis-4-(N-carbazolyl)phenyl)phenylphosphine oxide (BCPO), 10-(4-(4-(9H-carbazol-9-yl)phenylsulfonyl)phenyl)-9,9-dimethyl- 9,10-dihydroacridine (CzAcSF), and CBP were used. To achieve desirable emission, an additional emitter, 9,9′-((2-(4′-(9H-carbazol-9-yl)-[1,10-biphenyl]-4-yl)ethene-1,1-diyl)bis(4,1-phenylene))bis(9H-carbazole) (2CzTPEPCz), was used in tandem with emissive material EA20. To balance the flow of electrons, TBPi, TmPyPB, DPEPO, and B3PyPB were applied for electron-transporting layers. In order to make a lower electron injection barrier, LiF, Cs_2_CO_3_, or Liq were used for EILs in some cases as it can be seen in Table 8.

Various metrics of the OLEDs using EA derivatives are shown in Table 9. Green light emission of the D–A-type benzophenone derivatives was the most common result for 19 of the 28 single-emitter devices described in this section. There also were reported six blue, three yellow, and one white OLED prototype using materials of this group. The EQE exhibited by the devices ranged widely from 1.8% to 26.7%. The most effective yellow OLED device was achieved by Chen and colleagues and reached a maximum CE, PE, and EQE of 73.1 cd/A, 38.2 lm/W, and 26.7%, respectively [111]. For all the emitters used in yellow OLED devices, the TADF effect was confirmed. Zhao et al. successfully applied the same emitter EA20 in the white TADF OLED prototype D2EA20 and achieved a high CE of 45.9 cd/A, PE of 18.0 lm/W, and EQE of 20.8% [112]. Ho and co-workers synthesized and characterized triphenylethene-carbazole-substituted benzophenone derivative EA10. A big ΔE_ST_ gap of 1.09 eV could not enable the TADF process in this case. The green OLED device with DEA10 achieved an EQE of 1.7% and high luminance of 11,802 cd/m^2^. Ma et al. tested three different benzophenone-carbazole materials EA1, EA2, and EA3 with different positions of triphenylamine moiety in the structures. The most efficient TADF emitter was compound EA3, which in a green device DEA3, reached an external quantum efficiency of 7.6%. Kreiza and colleagues also achieved good results utilizing a benzophenone-based D–A TADF emitter EA12. Non-doped green OLED device D1EA12 achieved a CE, PE, and EQE of 19.0 cd/A, 14.9 lm/W, and 10.3%. By doping the mentioned emitter in a DPEPO host, characteristics were enhanced by the authors with 34.9 cd/A, 27.3 lm/W, and 12.5% for device D2EA12. Ma and co-workers presented another very efficient green light-emitting sulfone group-enriched benzophenone TADF emitter EA18. Luminance of the doped device D2EA18 surpassed 30,000 cd/m^2^ with an EQE reaching 20.6%. Other characteristics were also impressive with a CE reaching 64.6 cd/A and PE being 75.1 lm/W. Meanwhile, the similarly structured green TADF emitter EA17 obtained by the same researchers was less efficient in doped devices, which could be attributed to the absence of a sulfone group. However, it was impressive when applied in non-doped EMLs: device D1EA17 achieved a maximum PE, CE, and EQE of 53.7 cd/A, 52.7 lm/W, and 17.3%, respectively. In the realm of green light-emitting devices, the TADF emitter EA21, which had one of its benzophenone phenyl rings replaced with pyridine and was characterized by Wang and co-workers, demonstrated superior efficiency. Luminance of the doped device D2E21 surpassed 11,000 cd/m^2^, while its EQE was 25.6%. Other characteristics were also impressive with a CE and PE reaching 69.8 cd/A and 58.9 lm/W. The same green TADF emitter was also unrivalled when applied in non-doped EML. Device D1EA21 achieved a maximum PE, CE, and EQE of 56.4 cd/A, 43.5 lm/W, and 18.7%. These characteristics were considerably higher than those of devices D1EA15 and D2EA15, which used pyridine-unmodified green TADF emitter EA15. The benzophenone fragment was also successfully applied in the synthesis of blue TADF emitters. A group of researchers led by J. Wang successfully combined a benzophenone electron acceptor with the 11-phenyldihydroindolo[2,3-a]carbazole electron donor and obtained material EA6. When applied as a dopant in blue OLED DEA6, a maximum EQE of 17.7% and luminance of over 14,000 cd/m^2^ were obtained. Other efficiencies such as the CE and PE were 44.8 cd/A and 45.6 lm/W, respectively. However, the most efficient benzophenone derivative used as a blue TADF emitter was EA19, characterized by J. Zhang and his group of scientists. A relatively simple structure of a benzophenone-acridine derivative was applied for a blue DEA19 device and reached a CE of 47.7 cd/A, PE of 29.9 lm/W, and EQE of 20.6%

## 5. Benzophenone-Based Emitters Employing a Symmetric D–A–D Structure

**Scheme 4:**
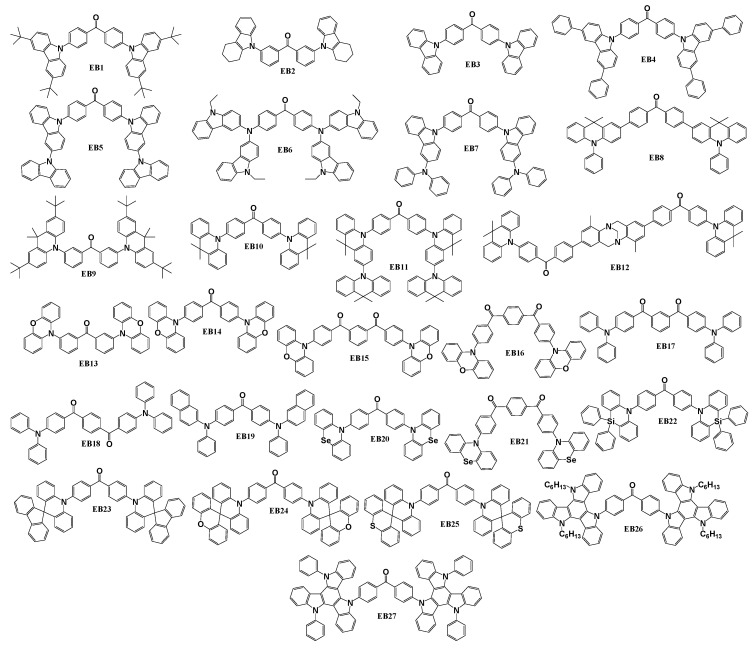
Structures of benzophenone-based symmetrical D–A–D materials used as emitters in OLEDs.

Chemical structures of symmetrical benzophenone-based emitters employing donor–acceptor–donor (D–A–D) structures are shown in Scheme 4. These compounds possess a molecular configuration comprising two separate donor segments enveloping a central acceptor moiety, establishing a conjugated system characterized by alternating donor and acceptor units. The materials described in this section have a mostly central benzophenone fragment with various carbazole substituents as in the case for compounds EB1–EB7. Acridine was also a popular fragment as an electron donor for five of the described materials EB8–EB12. Phenoxazine was a key element in four structures (EB13–EB16), and phenyl- or naphtyl- amines were used in three molecules, namely EB17–EB19. In addition, various other electron donors, such as spiro-, phenoselanazine, triazatruxene, and some others have been utilized. Material EB1 [113] was synthesized by utilizing Ullmann reaction between 4,4′-dibromobenzophenone and 3,6-bis(tert-butyl)-9H-carbazole. A significant number of the EB materials was synthesized through the Buchwald–Hartwig amination of 3,3′-dibromobenzophenone or 4,4′-dibromobenzophenone, employing various electron-donating fragments. For example, for the synthesis of compounds EB2, EB9, EB13 [78], and EB4 [81], tetrahydrocarbazole, phenoxazine, 2,7-ditert-butyl-9,9-dimethylacridine, and 3,6-diphenyl-9H-carbazole, respectively, served as the second reactant. The reactions of dibromobenzophenone with carbazole resulted in material EB3, with bicarbazole yielded EB5 [73], and with 9,9,9′9′-tetramethyl-9,9′10,10′-tetrahydro-2,10′-biacridine—EB11 [114]. N-(2-naphthyl)aniline played a crucial role in obtaining EB19 [115]. Phenoselanazine, azasiline, 10H-spiro[acridine-9,9′-fluorene], 10H-spiro[acridine-9,9′-xanthene], and (10H-spiro[acridine-9,9′-thioxanthene]) were utilized for the preparation of EB21 [116], EB22 [117], EB23, EB24, and EB25 [118], respectively. The synthesis of compounds EB26 and EB27 [119] involved the use of 5,10-dihexyl-10,15-dihydro-5H-diindolo[3,2-a:3′,2′-c]carbazole and 5,10-diphenyl-10,15-dihydro-5H-diindolo[3,2-a:3′,2′-c]carbazole as reactants. The synthesis of the benzophenone derivatives EB14, EB15, and EB16 [75], which was described in the same publication as for the earlier mentioned EB3 and EB5, was achieved using aminations of bis(4-bromobenzoyl)benzenes with two equivalents of phenoxazine. The same reaction methodology was used to obtain emitter EB6 [97]. In this case, amination of 3-iodo-9-ethylcarbazole with 4,4′-diaminobenzophenone took place. ((4,10-Dimethyl-6H,12H-5,11-methanodibenzo[b,f][1,5]diazocine-2,8-diyl)bis(4,1-phenylene))bis((4-bromophenyl)methanone) reacted with 9,9-dimethyl-9,10-dihydroacridine under similar conditions to prepare emitter EB12 [120]. Material EB7 [121] was synthesized during a simple nucleophilic substitution of 4,4′-diflourobenzophenone with 3-(N,N-diphenylamino)carbazole. Suzuki reaction was chosen as the best procedure for obtaining compound EB8 [122] from 4,4′-dibromobenzophenone and 9,9-dimethyl-10-phenyl-2-(4,4,5,5-tetramethyl-1,3,2-dioxaborolan-2-yl)-9,10-dihydroacridine as initial reactants. EB10 [123] was synthesized during the N,N-(2,6-di(3-pentyl)phenyl) imidazolium-catalysed reaction of 4,4′-dibromobenzophenone with 9,9-dimethyl-9,10-dihydroacridine. Two compounds EB17 and EB18 [88] were obtained through a simple one-step Friedel–Crafts reaction by using commercially available cheap starting materials triphenylamine (TPA) and, correspondingly, isophthaloyl dichloride for compound EB17 or terephthaloyl dichloride for emitter EB18.

Table 10 depicts thermal, electrochemical, and photophysical properties of the mentioned light-emitting symmetrical benzophenone materials EB1–EB27. The destruction temperatures were reported for eighteen compounds described in this section. Even though EB13 had a lowest T_D_ of 218 °C owing to two phenoxazine fragments, it was still high enough to cope with conditions of the device forming and operating, especially bearing in mind a high melting temperature of 118 °C for the compound. All the other presented emitters here were characterized by a T_D_ of 270 °C or higher. Glass transition temperatures for derivatives EB1, EB2, EB7, EB17, EB18, EB19, EB26, and EB27 were registered at 90 °C or higher, so these emitters could form stable amorphous layers. Crystalline benzophenone-based materials EB4, EB9, and EB22 demonstrated melting temperatures of 300 °C or higher as it was confirmed by DSC. Examining the HOMO–LUMO gap (energy bandgap E_g_), it becomes evident that the majority of the symmetrical D–A–D benzophenone derivatives exhibit measured or calculated E_g_ levels in the region of 2.47–3.37 eV. Only EB9 presented a distinctly elevated E_g_ level of 3.66 eV. This could be the reason of decreased quantum yield and less effective TADF processes, when compared to emitters possessing smaller bandgaps. Achieving a low ΔE_ST_ is essential in the development of an emitter to enhance the efficiency of the TADF process, as it is evident from the data of Table 10, where materials EB11, EB14, EB23, EB24, and EB25 with ΔE_ST_ values of 0.03 eV or lower demonstrate a Φ_PL_ exceeding 70% in a nitrogen atmosphere. The participation of the triplet state in the emission process was also demonstrated for some compounds. For example, this was observed in EB6, where the overall Φ_PL_ substantially decreased upon exposure to oxygen. In addition, in derivatives EB1, EB12, EB23, EB24, and EB25, substantial R_D_ levels were identified. All emitters described in this section, except fluorescent EB2 and EB9, exhibited TADF properties.

The majority of the D–A–D benzophenone-based derivatives were tested as emitters in OLEDs, whose structures are illustrated in Table 11. The dominant choice for anode in these devices was also ITO. To ease the energy barrier, such hole-injecting materials as Mo_2_O_3_, rhenium (VI) oxide (ReO_3_), and MoO_3_ were used. NPB, α-NPD, mCP, PEDOT:PSS, TAPC, HAT-CN, or TCTA were chosen for the formation of hole-transporting layers. To further elevate the efficiency of the devices, well-known host materials diphenyl[4-(triphenylsilyl)phenyl]phosphine oxide (TSPO1), DPEPO, TCzl, 9-(3-(9H-carbazol-9-yl)phenyl)-9H-carbazole-3-carbonitrile (mCPCN), 2,8-bis(diphenyl-phosphoryl)-dibenzo[b,d]furan (PPF), mCP, mCBP, CBP, and some others were used in emissive layers. To achieve desired white-light OLEDs, some phosphorescent emitters such as Ir(ppy)_2_(acac) or Ir(bt)_2_(dipba) were used in conjunction with benzophenone derivative EB4. Electron transport layers were made from DPEPO, TPBi, B3PYMPM, TmPyPB, BPhen, PPF, or TSPO1. Also, LiF, Liq, and rubidium carbonate (Rb_2_CO_3_) were used as electron-injecting materials. For all the devices described in this section, aluminium cathodes were used.

This section reviews all devices constructed by researchers utilizing benzophenone-based emitters EB1–EB27 with a D–A–D structure, and the characteristics of these devices are detailed in Table 12. The combination of a central benzophenone electron-accepting group with various electron donors yielded red, yellow, green, and blue emitters. Utilization of the described compounds with phosphorescent emitters or other benzophenone-based materials also resulted in white light emission. Lee et al. reported the red-emitting material EB15. Device DEB15, which utilized the mentioned TADF emitter, exhibited a low turn-on voltage of 2.8 V, CE of 11.1 cd/A, and EQE of 4.2%. Additionally, maximum luminance of the device exceeded 50,000 cd/m^2^. The same authors introduced blue TADF emitters EB3 and EB5, which in devices achieved EQE values of 8.1% and 14.3%, respectively, with the latter one being the most efficient blue OLED in this D–A–D group. They also presented a green TADF emitter EB14, which attained an EQE of 10.7% in its device, and a yellow TADF emitter EB16, which reached an EQE of 6.9%. Liang and co-workers presented material EB4 which can act as a host and as an emitter at the same time. For example, device D1EB4 with non-doped emitter EB4 demonstrated a PE and EQE of 6.9 lm/W and 4.0%, respectively. By combining phosphorescent emitters Ir(ppy)_2_(acac) and Ir(bt)_2_(dipba) with the compound EB4, device D2EB4 achieved white emission and demonstrated an exceptionally high current efficiency of 48.6 cd/A and external quantum efficiency of 25.6%. By utilizing the symmetrical D–A–D structure, researchers developed nine green TADF emitters EB6, EB10, EB11, EB14, EB20, EB21, EB23, EB24, and EB25, which achieved over 10% external quantum efficiency. EB6, designed by Tani and colleagues, was used in an emissive layer of the TADF device DEB6 and achieved an EQE of 10.4%. Zhang and collaborators employed a straightforward structural derivative EB10 as a non-doped TADF emitter. The resultant device exhibited excellent PE and EQE values of 59.0 lm/W and 18.0%, respectively. Liu and colleagues created a structurally similar derivative EB11, where they replaced the acridine electron donor with a biacridine fragment. This modification led to a notable improvement in the EQE, reaching 22.5%, along with an impressive CE of 69.3 cd/A. Derivatives EB20 and EB21, which were characterized by Sharif and co-workers, also were highly efficient when applied in green TADF OLED devices. By combining phenoselenazine electron donors with benzophenone, researchers synthesized EB20 and fabricated the DEB20 device, which exhibited an exceptionally high EQE of 30.8% and a CE of 64.0 cd/A. In comparison, the DEB21 device, which employed derivative EB21 with an additional ketone group as the emitter, achieved a lower EQE of 18.8% but with a higher CE of 73.5 cd/A. The highest overall efficiencies of green TADF OLEDs by utilizing symmetrical D–A–D benzophenone-based emitters were achieved by Huang et al., who synthesized and characterized derivatives EB23, EB24, and EB25. These materials underwent testing in both non-doped and doped emissive layers. The most efficient non-doped device D1EB23 demonstrated exceptional performance with a maximal CE, PE, and EQE reaching 53.9 cd/A, 48.9 lm/W and 18.6%, respectively. In the doped device D2EB23, efficiencies were further elevated, achieving an impressive CE of 90.9 cd/A, PE of 91.2 lm/W, and EQE of 30.3%. If the device D2EB23 attained the highest CE and PE values among the green devices described in this section, the OLED D2EB24 with emitter EB24 doped in a host material remained unparalleled with an extraordinary high EQE level of 32.2%.

Within the realm of blue TADF emitters described in this section, aside from the previously mentioned derivatives EB3 and EB5, notable efficiencies were also observed by using EB8 and EB22 emitters in TADF-based OLEDs. The compound EB8, presented by Cai and colleagues, achieved an EQE of 8.90% in the DEB8 device. Another team led by Sun developed and utilized the TADF emitter EB22 as crucial element in the construction of the DEB22 device, displaying a V_ON_ of 3.6 V, an L_MAX_ of 2021 cd/m^2^, and an EQE of 11.4%. The previously mentioned EB5 emitter proved to be the most effective blue TADF emitter in this section. When integrated into a device, it exhibited a CE of 25.5 cd/A, an EQE of 14.3%, and a maximum luminance of 3900 cd/m^2^.

## 6. Benzophenone-Based TADF Emitters Employing an Asymmetric D–A–D Structure

**Scheme 5:**
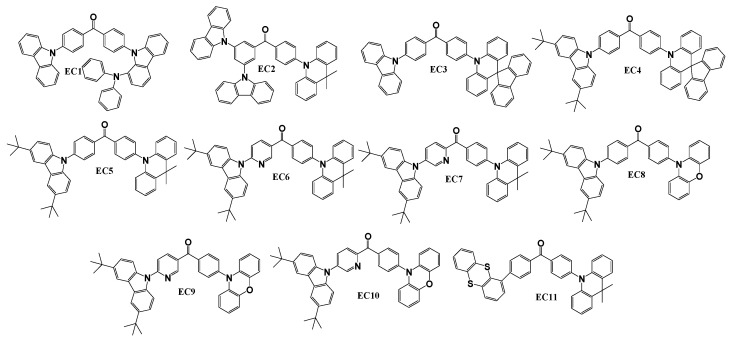
Structures of benzophenone-based asymmetrical D–A–D materials used as emitters in OLEDs.

Structures of benzophenone-based TADF emitters having asymmetric D–A–D molecular configurations are shown in Scheme 5. Except for one material, all others utilized carbazol-9-yl as one of the electron-donating fragments in conjunction with a benzophenone acceptor, along with other donating moieties like 3-substituted carbazole, 9,10-dihydro-9,9-dimethylacridine, spiro[acridine-9,9′-fluorene], or phenoxazine. Only the derivative EC11 did not incorporate carbazole as one of the electron donors, but employed 9,10-dihydro-9,9-dimethylacridine and thianthrene fragments. To obtain the derivatives presented in this section, at least two synthetic steps were required. Derivative EC1 [94] was synthesized through a two-step Buchwald–Hartwig amination procedure, involving the replacement of one bromine atom in 4,4′-dibromobenzophenone with carbazole by following reaction with 4-(9H-carbazol-1-yl)-N,N′-diphenylaniline. Material EC2 [66] was obtained by reaction between (3,5-bis-carbazol-9-yl-phenyl)-(4-bromophenyl)-methanone and 9,10-dihydro-9,9-dimethylacridine under Buchwald–Hartwig reaction conditions. Derivatives EC3 and EC4 [124] were created through the two-step synthesis process. Initially, the fluorine atom of 4-bromo-4′-fluorobenzophenone was reacted with spiro[acridine-9,9′-fluorene] using a nucleophilic substitution reaction. Subsequently, the bromine atom was replaced with carbazole or 3,6-di-tert-butylcarbazole during Buchwald–Hartwig reaction. Materials EC5–EC10 were synthesized using very similar procedures [125]. In the initial step, 3,6-di-tert-butyl-9H-carbazole underwent Buchwald–Hartwig reaction with (4-bromophenyl)(4-fluorophenyl)methanone, (6-bromopyridin-3-yl)(4-fluorophenyl)methanone, or (5-bromopyridin-2-yl)(4-fluorophenyl)methanone, resulting in intermediate compounds. Subsequently, nucleophilic substitution reactions of 9,10-dihydro-9,9-dimethylacridine were employed with, correspondingly, (4-(3,6-di-tert-butyl-9H-carbazol-9-yl)phenyl)(4-fluorophenyl)methanone, (6-(3,6-di-tert-butyl-9H-carbazol-9-yl)pyridin-3-yl)(4-fluorophenyl)methanone, and (5-(3,6-di-tert-butyl-9H-carbazol-9-yl)pyridin-2-yl)(4-fluorophenyl)methanone, to yield compounds EC5, EC6, and EC7, respectively. Utilizing the same reactions with phenoxazine instead of 9,10-dihydro-9,9-dimethylacridine resulted in compounds EC8, EC9, and EC10, respectively. The synthesis of derivative EC11 also involved a two-step process [126]. Initially, one bromine atom of 4,4′-dibromobenzophenone was replaced with a thianthrene moiety through a Suzuki reaction. Subsequently, the second bromine atom was substituted with 9,10-dihydro-9,9-dimethylacridine in a Buchwald–Hartwig cross-coupling reaction.

Table 13 presents the thermal, electrochemical, and photophysical properties of materials EC1–EC11. The asymmetric D–A–D-type benzophenone derivatives exhibited exceptional thermal stability, with a T_D_ ranging from 309 to 451 °C, as verified through TGA measurements. It is reported that for materials EC2 and EC11, during DSC experiments, T_G_ values were registered at 72 °C and 104 °C, respectively. The HOMO levels of materials EC1–EC11 ranged from −5.92 to −5.23, while the LUMO levels varied between −3.09 and −2.61. The energy difference between HOMO and LUMO levels for all the materials discussed in this section was 2.88 eV or lower. This small bandgap facilitates a minimal energy difference between the lowest singlet and triplet states, enabling efficient reverse intersystem crossing. Experimental results support this, with the ΔE_ST_ being 0.10 eV or lower, resulting in a high Φ_PL_ ranging from 33.2% to 90.0%. EC3 to EC11 derivatives exhibited aggregation-induced emission properties with lower Φ_PL_ values detected in solutions compared to film states. Additionally, materials EC1 and EC3 to EC10 demonstrated significant involvement of triplet states in photon generation, as evidenced by a R_D_ ranging from 33.0% to 89.7% with one of the potential light-generating mechanisms being TADF.

Every benzophenone derivative presented in this section underwent testing as an emitter in OLED devices, with their structures depicted in Table 14. Consistent with earlier sections, ITO was the only selection for the anode in these devices. To reduce the energy barrier for holes, the device DEC11 utilized MoO_3_ as the hole-injecting material. For this device or others, a stack of one to three layers of hole-transporting materials was employed, featuring layers composed of PEDOT:PSS, HAT-CN, TAPC, TCTA, NPB, or mCP. While most devices using EC1-EC11 emitters employed non-doped configurations, some of them also utilized the host–guest approach. Specifically, the host material CBP was applied in device DEC2, and PPF was employed for D2EC3 and D2EC4. TPBi, TmPyPb, and PPF were employed as electron-transporting layers, while Cs_2_CO_3_ and LiF served as electron-injecting materials. For all the devices discussed in this section, the Al cathode was the only option.

All the recently presented derivatives EC1–EC11 underwent testing as emitters in devices and the characteristics of these OLEDs are detailed in Table 15. Although most of these devices displayed green emission, there were exceptions such as DEC8, which emitted yellow light, and DEC9 as well as DEC10, which emitted orange light. Ma and co-workers successfully synthesized and characterized an EC1 derivative, revealing noteworthy characteristics. The mentioned emitter was integrated into the non-doped emissive layer of device DEC1, which demonstrated a CE of 35.5 cd/A, PE of 22.3 lm/W, and EQE of 13.3%. Another derivative EC2, synthesized and characterized by Zhao et al., showcased impressive performance with CE, PE, and EQE values of 61.8 cd/A, 40.4 lm/W, and 19.7%, respectively, as well as with an impressive L_MAX_ of 116,000 cd/m^2^. Huang’s team developed derivatives EC3 and EC4. Among them, TADF emitter EC3-based devices exhibited the highest efficiencies between devices described in this section, achieving a CE, PE, and EQE of 76.9 cd/A, 71.0 lm/W and 29.0%, respectively, in the non-doped device D1EC3. Introducing host material PPF in the emissive layer of the D2EC3 further elevated efficiencies to 82.9 cd/A for CE, 70.1 lm/W for PE, and a peak EQE reaching 33.3%. Although EC4 using OLEDs were slightly less efficient, D1EC4 exhibited a remarkably low V_ON_ of 3.0 V and EQE of 21.6%. D2EC4 demonstrated an impressive EQE of 32.9%, with CE and PE values of 77.2 cd/A and 65.0 lm/W, respectively. The emitting materials EC5–EC10 were meticulously designed, synthesized, and tested by Ma and colleagues in non-doped OLED prototypes. These materials combined a 3,6-di-tert-butylcarbazole donor with various other donors such as 9,10-dihydro-9,9-dimethylacridine (EC5, EC6, EC7) or phenoxazine (EC8, EC9, EC10) with acceptors like benzophenone (EC5, EC8), phenyl(3-pyridyl)methanone (EC6, EC9), or phenyl(2-pyridyl)methanone (EC7, EC10). For instance, the incorporation of 9,10-dihydro-9,9-dimethylacridine moiety in benzophenone-based TADF emitter EC5 led to the device DEC5 having a PE, CE, and EQE of 14.3 cd/A, 6.4 lm/W, and 6.70%, respectively. The incorporation of a pyridinyl fragment in TADF emitter EC6 significantly improved efficiencies in its corresponding device DEC6, reaching a CE of 35.4 cd/A, PE of 15.9 lm/W, and EQE of 11.4%. A phenoxazine fragment in benzophenone derivative EC8 resulted in a yellow TADF OLED device that achieved an EQE of 4.8%. An introduction of the pyridinyl fragment in compound EC9 elevated the efficiency of the orange TADF device DEC9, demonstrating a CE, PE, and EQE values of table21.6 cd/A, 6.80 lm/W, and 9.40%, respectively. Finally, the last TADF emitter of the group EC11, designed and synthesized by Tomkeviciene and colleagues, featured two electron-donating fragments of thianthrene and 9,10-dihydro-9,9-dimethylacridine. When incorporated into the emissive layer of device DEC11, CE, PE, and EQE values of 57.8 cd/A, 38.8 lm/W, and 22.2% with an L_MAX_ exceeding 15,000 cd/m^2^ were achieved.

## 7. Benzophenone-Based TADF Emitters Employing a Dendritic Structure

**Scheme 6:**
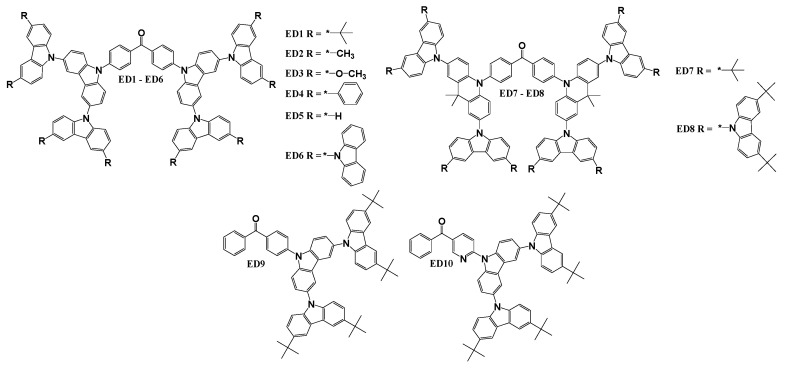
Structures of benzophenone-based dendritic materials used as emitters in OLEDs.

The structures of dendritic benzophenone-based TADF emitters are illustrated in Scheme 6. In the case of materials ED9 and ED10, researchers employed a donor–acceptor (D–A) dendritic structure, while all other materials featured donor–acceptor–donor (D–A–D) dendritic structures. The benzophenone as an electron acceptor was paired with either 9,10-dihydro-9,9-dimethylacridine or 9H-carbazole. Additionally, these electron donors were further substituted with diverse alkyl or aryl groups. Material ED1 [113] was synthesized during Ulmann reaction between 3,6-bis(3,6-di-tert-butylcarbazol-9-yl)carbazole and 4,4′-dibromobenzophenone. ED2, ED3, and ED4 [127] derivatives were acquired through N-arylation reactions involving 4,4′-diiodobenzophenone. The reaction with 3,6-bis(3,6-dimethylcarbazol-9-yl)carbazole resulted in ED2, while ED3 was obtained by using 3,6-bis(3,6-dimethoxycarbazol-9-yl)carbazole, and ED4 was derived in the reaction with 3,6-bis(3,6-diphenylcarbazol-9-yl)carbazole. ED5 and ED6 [128] compounds were synthesized using a closely related procedure but with distinct carbazole-based reactants. Specifically, 3,6-bis(carbazol-9-yl)carbazole was employed to produce ED5, while ED6 was derived from 3,6-bis(3,6-di(carbazol-9-yl)carbazol-9-yl)carbazole. For the synthesis of derivatives ED7 and ED8 [129], Ulmann reactions were employed with bis(4-(2,7-diiodo-9,9-dimethylacridin-10(9H)-yl)phenyl)methanone. The reaction with 3,6-di-tert-butylcarbazole resulted in ED7, and the reaction with 3,6-bis(3,6-di-tert-butylcarbazol-9-yl)carbazole produced material ED8. Compounds ED9 and ED10 [108] were also synthesized through the Ullmann reaction procedure, when 3,6-bis(3,6-di-tert-butylcarbazol-9-yl)carbazole reacted with the 4-bromobenzophenone to yield material ED9 and with (6-bromopyridin-3-yl)(phenyl)methanone to yield derivative ED10.

The thermal, electrochemical, and photophysical characteristics of the emitting materials ED1–ED10 are presented in Table 16. The dendritic benzophenone derivatives displayed remarkable thermal stability with the measured T_D_ for the tested materials exceeding 470 °C. In the cases of materials ED1, ED7, and ED8, DSC experiments confirmed only a T_G_ of 218 °C, 283 °C, and 289 °C, respectively. The HOMO levels for materials EC1–EC11 ranged from −5.87 to −5.04, while the LUMO levels varied between −3.09 and −2.03. The energy gap between the HOMO and LUMO levels for all discussed materials in this section was lower than 3.12 eV. This narrow gap facilitates a minimal energy difference between the lowest singlet and triplet states, promoting efficient reverse intersystem crossing. Experimental findings corroborated this, with a ΔE_ST_ of 0.15 eV or lower, and a high Φ_PL_ of up to 77.0% was obtained. Specifically, derivatives ED1, ED4, ED5, and ED6 exhibited aggregation-induced emission properties with lower Φ_PL_ values observed in solutions as compared to film states. Furthermore, materials ED1–ED6, ED9, and ED10 demonstrated the involvement of triplet states in photon generation, as indicated by the R_D_ ranging from 9.0% to 64.5%. One of the potential light-generating mechanisms could be TADF in the case.

All in this section, the described dendritic benzophenone derivatives underwent testing as emitters in OLED devices, and their structures are illustrated in Table 17. As in previous sections, ITO was consistently chosen as the anode material for these devices. The devices utilized one or a stack of two layers of hole-transporting materials, incorporating layers made of PEDOT:PSS, poly(9-vinylcarbazole) (PVK), TAPC, or mCP. While most devices incorporating emitters ED1-ED10 adopted non-doped configurations, devices D2ED9 and D2ED10 employed the host–guest approach with the host material DPEPO. The electron-transporting layers featured TPBi, TmPyPb, 2,7-bis(diphenylphosphoryl)-9,9′-spirobi[fluorene] (SPPO13), and DPEPO, while Cs_2_CO_3_, calcium (Ca), Liq, or LiF were employed as electron-injecting materials. In all the devices discussed in this section, the exclusive choice for the cathode was Al.

Table 18 presents the characteristics of OLED devices utilizing emitters ED1–ED10. Consistent with preceding sections, most of these devices emitted a green light, except for the yellow-emitting OLED with methoxy-substituted derivative ED3 and the blue-emitting device with derivative ED9 employing a D–A structure. Material ED1 exhibited a CE and EQE of 9.2 cd/A and 4.3%, respectively, in device D1ED1. Matsuoka et al. further explored this TADF derivative, optimizing layer structures in device D2ED1 to achieve the highest CE, PE, and EQE of 46.6 cd/A, 40.7 lm/W, and 17%, respectively, between non-doped devices of this section. The same research team also synthesized and characterized derivatives ED2, ED3, and ED4. Green devices DED2 and DED4 were less efficient than D2ED2 with EQEs of 9.0% and 8.8%, respectively. This difference may be attributed to substituting groups of outermost carbazoles in the structures. The only yellow device, DED3 of this section, demonstrated a CE of 17.7 cd/A, PE of 19.0 lm/W, and EQE of 6.4%. Matsuoka and team investigated dendritic TADF materials ED5 and ED6 [130]. When applied in emissive layers, emitter ED5 proved more effective than the additional carbazole fragments having derivative ED6. Device DED5 reached a maximum CE, PE, and EQE of 14.0 cd/A, 11.5 lm/W, and 5.7%, respectively. Li and co-workers developed and tested new benzophenone-acridine TADF cored dendritic materials ED7 and ED8. The derivative ED7, with fewer carbazole fragments, proved more efficient, reaching a maximum EQE of 12.0% compared to 5.20%, demonstrated by device DED8. Moreover, device DED7 exhibited an L_MAX_ of over 10,000 cd/m^2^. Wang et al. designed and synthesized D–A-type carbazole-dendronized TADF emitting materials ED9 and ED10. Non-doped emitter ED9, utilized in the blue device D1ED9, achieved a CE, PE, and EQE of 9.70 cd/A, 6.10 lm/W, and 4.2%, respectively. Doping ED9 in host material DPEPO significantly increased the efficiency of D2ED9, demonstrating a CE of 28.5 cd/A, PE of 24.9 lm/W, and EQE of 13.4%. Compound ED10, designed by changing the benzophenone electron acceptor with 3-benzoylpyridine, proved more efficient. In a non-doped configuration, device D1ED10 achieved a CE, PE, and EQE of 24.0 cd/A, 15.6 lm/W, and 8.50%, respectively. By introducing host material DPEPO in the emissive layer, the highest efficiency described in this section was achieved in D2ED10, demonstrating CE of 44.4 cd/A, PE of 42.8 lm/W, and EQE of 18.9% for the green-blue device.

## 8. Concluding Remarks

This review delves into the recent advancements in electroactive materials derived from benzophenone, providing meticulous attention to their synthesis and physical properties and the performance of organic light-emitting diodes incorporating these derivatives. Benzophenone-based materials exhibit versatile roles, serving as host materials for phosphorescent or TADF emitters, as well as functioning as emitting materials, including those with a TADF effect. A number of derivatives based on benzophenone have proven highly efficient as host materials for phosphorescent emitters, significantly improving the quantum efficiency and reducing the driving voltage of the organic light-emitting devices. Notably, benzophenone materials featuring two 3,6-diphenylcarbazole fragments have demonstrated an exceptional effectiveness for red, orange, and green phosphorescent OLEDs (PhOLEDs), achieving respective EQEs of 22.1%, 23.1%, and 25.1%. For blue phosphorescent emitters, the most suitable host exhibited a donor–acceptor (D–A) structure, incorporating one dimethylcarbazole fragment and achieving a device with an EQE of 19.4%. In the realm of green TADF materials, benzophenone derivatives with two 3-(4-(9H-carbazol-9-yl)phenyl)-9H-carbazole fragments emerged as highly effective hosts, resulting in a device with an EQE of 25.3%. Additionally, the substitution of benzophenone with N-phenyl-1-naphthylamine enabled the creation of a TADF host-emitter, successfully applied to a white OLED device with an EQE of 9.5%. Substituted benzophenones as light-emitting materials span the spectrum of light emission ranging from red to blue, which is achieved through the modification and incorporation of various moieties into the benzophenone backbone. Among the array of benzophenone-based TADF materials, derivatives employing donor–acceptor (D–A) structures proved highly effective as emitters for green and yellow TADF OLEDs, boasting EQE levels of 25.6% and 26.7%, respectively. This structural type demonstrated optimal suitability for application as blue TADF materials with acridine-substituted benzophenone serving as an emitter for blue TADF OLEDs, achieving an impressive EQE of 20.6%. In the case of symmetrical D–A–D structure benzophenone-based TADF emitters, their EQE reached 14.3% for blue and a remarkable 32.2% for green devices. The efficiency of green devices was further enhanced with asymmetrical D–A–D structured benzophenones by incorporating carbazole and 10H-spiro[acridine-9,9’-fluorene] electron donors. A derivative with this emitter demonstrated a device EQE of 33.3%. Researchers also aimed to achieve stable, long-lasting, non-doped TADF OLED devices by designing carbazole-dendronized benzophenone derivatives. The most efficient green OLED of this type exhibited an EQE of 17.0%. Therefore, benzophenones substituted with various electron donors are promising as emitters and hosts for various configurations of OLED devices, and further research in the field of new benzophenone-based electroactive materials is actively ongoing in order to improve the characteristics of future OLED devices.

## Figures and Tables

**Table 1:** Thermal, electrochemical, photoelectrical, and photophysical properties of materials HA1–HA10.

	T_M_, °C	T_G_, °C	T_Cr_, °C	T_D_, °C	E_g_, eV	E_S_, eV	E_T_, eV	ΔE_ST_, eV	HOMO, eV	LUMO, eV	Φ_PL_ Film, %	Φ_PL_ Sol., %
HA1	–	91	–	312	3.21	–	3.02	–	−5.17	−2.03	20	3
HA2	118	–	62	218	3.05	–	2.65	–	−4.74	−2.19	15	12
HA3	342	–	269	389	3.66	2.70	2.66	0.04	−4.78	−2.02	16	6
HA4	–	188	–	553	3.00	–	2.61	–	−5.80	−2.80	–	–
HA5	370	–	–	480	3.52	2.83	2.69	0.14	−5.77	−2.25	24.4	15.3
HA6	–	131	–	–	3.10	3.70	3.00	0.70	−5.60	−2.50	–	–
HA7	–	55	–	339	2.72	2.77	2.53	0.24	−5.51	−2.79	–	–
HA8	272	–	–	337	3.91	–	2.97	–	−5.80	−1.89	–	–
HA9	291	–	–	445	3.93	–	2.97	–	−5.76	−1.83	–	–
HA10	–	122	–	466	3.91	–	2.95	–	−5.78	−1.87	–	–

**Table 2:** Architectures of devices utilizing host materials HA1–HA10.

Device with HA Host	Device Architecture
DHA3	ITO/TAPC (50 nm)/mCP (10 nm)/**HA3**:7 wt% Ir(piq)_2_acac (30 nm)/Bphen (50 nm)/LiF (1 nm)/Al (100 nm)
DHA4	ITO (130 nm)/PEDOT:PSS (30 nm)/TFB (20 nm)/**HA4**:8 wt% Ir(ppy)_3_/Cs_2_CO_3_:Al (100 nm)
D1HA5	ITO/NPB (35 nm)/mCP (5 nm)/**HA5**:3 wt% Ir(ppy)_2_acac (30 nm)/B3PYMPM (30 nm)/LiF (0.5 nm)/Al (150 nm)
D2HA5	ITO/NPB (35 nm)/mCP (5 nm)/**HA5**:3 wt% Ir(bzq)2(dipba) (30 nm)/B3PYMPM (30 nm)/LiF (0.5 nm)/Al (150 nm)
D3HA5	ITO/NPB (35 nm)/mCP (5 nm)/**HA5**:3 wt% Ir(bt)2(dipba) (30 nm)/B3PYMPM (30 nm)/LiF (0.5 nm)/Al (150 nm)
D1HA6	ITO/TAPC (30 nm)/TCTA (10 nm)/**HA6**:FIrpic (20 nm)/CzPhPy (10 nm)/TmPyPB (45 nm)/LiF/Al
D2HA6	ITO/TAPC (30 nm)/TCTA (10 nm)/**HA6**:Ir(ppy)_2_(acac) (20 nm)/CzPhPy (10 nm)/TmPyPB (45 nm)/LiF/Al
D3HA6	ITO/TAPC (30 nm)/TCTA (10 nm)/**HA6**: Ir(mphmq)_2_(tmd) (20 nm)/HA8 (10 nm)/CzPhPy (10 nm)/TmPyPB (45 nm)/LiF/Al
DHA7	ITO/HAT-CN (10 nm)/TAPC (65 nm)/TCTA (10 nm)/**HA7**:10 wt% Ir(MDQ)2acac (30 nm)/TmPyPb (55 nm)/Liq (1 nm)/Al (110 nm)
D1HA8	ITO/NPB (40 nm)/mCP (10 nm)/**HA8**:9–10 wt% Ir(ppy)_3_ (30 nm)/TmPyPB (45 nm)/LiF (2 nm)/Al (150 nm)
D2HA8	ITO/NPB (40 nm)/mCP (10 nm)/**HA8**:9–10 wt% Ir(btp)_2_acac (30 nm)/TmPyPB (45 nm)/LiF (2 nm)/Al (150 nm)
D3HA8	ITO/NPB (40 nm)/mCP (10 nm)/**HA8**:9–10 wt% FIrpic (30 nm)/TmPyPB (45 nm)/LiF (2 nm)/Al (150 nm)
D4HA8	ITO/NPB (40 nm)/mCP (10 nm)/**HA8**:9–10 wt% PO-01 (30 nm)/TmPyPB (45 nm)/LiF (2 nm)/Al (150 nm)
D1HA9	ITO/NPB (40 nm)/mCP (10 nm)/**HA9**:9–10 wt% Ir(ppy)_3_ (30 nm)/TmPyPB (45 nm)/LiF (2 nm)/Al (150 nm)
D2HA9	ITO/NPB (40 nm)/mCP (10 nm)/**HA9**:9–10 wt% Ir(btp)_2_acac (30 nm)/TmPyPB (45 nm)/LiF (2 nm)/Al (150 nm)
D3HA9	ITO/NPB (40 nm)/mCP (10 nm)/**HA9**:9–10 wt% FIrpic (30 nm)/TmPyPB (45 nm)/LiF (2 nm)/Al (150 nm)
D4HA9	ITO/NPB (40 nm)/mCP (10 nm)/**HA9**:9–10 wt% PO-01 (30 nm)/TmPyPB (45 nm)/LiF (2 nm)/Al (150 nm)
D1HA10	ITO/NPB (40 nm)/mCP (10 nm)/**HA10**:9–10 wt% Ir(ppy)_3_ (30 nm)/TmPyPB (45 nm)/LiF (2 nm)/Al (150 nm)
D2HA10	ITO/NPB (40 nm)/mCP (10 nm)/**HA10**:9–10 wt% Ir(btp)_2_acac (30 nm)/TmPyPB (45 nm)/LiF (2 nm)/Al (150 nm)
D3HA10	ITO/NPB (40 nm)/mCP (10 nm)/**HA10**:9–10 wt% FIrpic (30 nm)/TmPyPB (45 nm)/LiF (2 nm)/Al (150 nm)
D4HA10	ITO/NPB (40 nm)/mCP (10 nm)/**HA10**:9–10 wt% PO-01 (30 nm)/TmPyPB (45 nm)/LiF (2 nm)/Al (150 nm)

**Table 3:** Characteristics of phosphorescent OLED devices using host materials HA1–HA10.

Device	Host	Colour	V_ON_, V	L_MAX_, cd/m^2^	CE, cd/A	PE, lm/W	EQE, %	CIE (x, y)
DHA3	HA3	Red	–	1094	5.6	5.30	8.60	(0.68, 0.32)
DHA4	HA4	Green	–	–	–	22.0	14.6	–
D1HA5	HA5	Green	2.5	93,330	–	99.1	25.1	(0.29, 0.64)
D2HA5	HA5	Orange	2.6	31,200	–	61.6	23.1	(0.51, 0.47)
D3HA5	HA5	Red	2.9	10,240	–	27.1	22.1	(0.61, 0.36)
D1HA6	HA6	Blue	3.0	–	–	38.2	19.4	(0.16, 0.33)
D2HA6	HA6	Green	2.9	–	–	75.7	21.0	(0.29, 0.64)
D3HA6	HA6	Red	3.1	–	–	30.8	16.5	(0.62, 0.38)
DHA7	HA7	Red	4.0	3876	20.6	12.6	16.1	–
D1HA8	HA8	Green	4.5	3080	46.8	29.1	17.0	(0.30, 0.60)
D2HA8	HA8	Red	5.0	905	5.5	2.2	5.9	(0.65, 0.32)
D3HA8	HA8	Blue	5.5	708	10.9	4.6	5.3	(0.17, 0.32)
D4HA8	HA8	Yellow	5.5	3490	50.6	28.9	19.2	(0.47, 0.51)
D1HA9	HA9	Green	4.5	4940	45.6	31.9	15.6	(0.31, 0.60)
D2HA9	HA9	Red	4.5	1840	13.2	9.2	10.8	(0.67, 0.33)
D3HA9	HA9	Blue	4.5	1010	9.3	3.7	4.5	(0.17, 0.32)
D4HA9	HA9	Yellow	4.5	6480	50.8	31.9	18.5	(0.48, 0.51)
D1HA10	HA10	Green	4.5	5020	37.5	26.2	12.1	(0.31, 0.60)
D2HA10	HA10	Red	4.0	1820	19.9	15.6	13.7	(0.67, 0.33)
D3HA10	HA10	Blue	5.5	473	8.5	4.9	4.0	(0.19, 0.32)
D4HA10	HA10	Yellow	4.5	10,700	30.5	21.3	10.7	(0.48, 0.52)

**Table 4:** Thermal, electrochemical, photoelectrical, and photophysical properties of materials HB1–HB8.

	T_M_, °C	T_G_, °C	T_Cr_, °C	T_D_, °C	E_g_, eV	E_S_, eV	E_T_, eV	ΔE_ST_, eV	HOMO, eV	LUMO, eV	Φ_PL_ Film, %	Φ_PL_ Sol., %	
												N_2_	O_2_
HB1	–	90	–	436	2.84	2.59	2.38	0.21	−5.31	−2.47	75	46	–
HB2	–	92	–	416	2.70	2.37	2.32	0.05	−5.32	−2.62	2	2	–
HB3	218	101	203	411	2.89	3.07	2.55	0.52	−5.13	−1.47	9	9	–
HB4	241	107	–	428	2.78	3.00	2.53	0.47	−5.08	−1.53	13	13	–
HB5	–	156	–	–	3.33	2.91	2.61	0.30	−5.38	−2.05	–	–	–
HB6	123	92	–	277	4.10	2.97	2.64	0.33	−6.15	−2.63	–	–	–
HB7	366	187	–	371	4.00	2.95	2.60	0.35	−6.05	−2.45	–	–	–
HB8	–	–	–	497	2.80	2.61	2.60	0.01	−5.35	−2.55	–	8	6

**Table 5:** Architectures of devices utilizing host materials HB1–HB8.

Device	Device Architecture
DHB1	ITO/PEDOT:PSS (40 nm)/**HB1**:10 wt% 4CzCNPy (35–40 nm)/TmPyPB (60 nm)/LiF (0.8 nm)/Al (120 nm)
DHB2	ITO/PEDOT:PSS (40 nm)/**HB2**:10 wt% 4CzCNPy (35–40 nm)/TmPyPB (60 nm)/LiF (0.8 nm)/Al (120 nm)
DHB3	ITO/MoO_3_ (8 nm)/NPB (60 nm)/TAPC (5 nm)/**HB3**:5 wt% 4CzTPN (5 nm)/**HB3** (15 nm)/ PFBP-2b(20 wt%):TPBi (40 nm)/LiF (1 nm)/Al
DHB4	ITO/MoO_3_ (8 nm)/NPB (60 nm)/TAPC (5 nm)/**HB4**:5 wt% 4CzTPN (5 nm)/**HB4**(15 nm)/ PFBP-2b(20 wt%):TPBi (40 nm)/LiF (1 nm)/Al
DHB5	ITO (125 nm)/PEDOT:PSS (30 nm)/**HB5**:15 wt% 4CzIPN (20 nm)/TPBi (30 nm)/LiF (1.0 nm)/Al (150 nm)
D1HB6	ITO (125 nm)/PEDOT:PSS (35 nm)/**HB6**:4CzIPN (20 nm)/PO-T2T (10 nm)/TPBi (30 nm)/LiF (1 nm)/Al (200 nm)
D2HB6	ITO (125 nm)/PEDOT:PSS (35 nm)/VPEC (10 nm)/**HB6**:4CzIPN (20 nm) /PO-T2T (10 nm)/TPBi (30 nm)/LiF (1 nm)/Al (200 nm)
DHB7	ITO (125 nm)/PEDOT:PSS (35 nm)/**HB7**:4CzIPN (20 nm)/PO-T2T (10 nm)/TPBi (30 nm)/LiF (1 nm)/Al (200 nm)
DHB8	ITO/PEDOT:PSS (40 nm)/**HB8**:10 wt% 4CzCNPy (40 nm)/TmPyPB (60 nm)/LiF (0.8 nm)/Al (100 nm)

**Table 6:** Characteristics of TADF OLED devices using host materials HB1–HB8.

Device	Host	Colour	V_ON_, V	L_MAX_, cd/m^2^	CE, cd/A	PE, lm/W	EQE, %	CIE (x, y)
DHB1	HB1	Green	3.5	20,322	43.5	33.3	13.0	(0.32, 0.60)
DHB2	HB2	Green	3.2	15,510	29.3	25.4	9.0	(0.33, 0.58)
**DHB3**	**HB3**	**White**	**3.9**	**29,922**	**18.6**	–	**9.5**	**(0.36, 0.31)**
DHB4	HB4	White	3.6	15,350	13.8	–	7.1	(0.32, 0.31)
DHB5	HB5	Green	3.4	8601	9.5	8.4	2.8	(0.31, 0.59)
D1HB6	HB6	Green	3.0	16,500	70.7	55.6	23.2	(0.28, 0.57)
**D2HB6**	**HB6**	**Green**	**2.9**	**18,900**	**72.3**	**63.6**	**25.3**	**(0.29, 0.58)**
DHB7	HB7	Green	2.7	10,540	49.2	46.2	15.3	(0.28, 0.57)
DHB8	HB8	Green	3.7	22,004	38.3	–	12.5	(0.34, 0.58)

Characteristics of best performing white and green devices are highlighted in bold.

**Table 7:** Thermal, electrochemical, photoelectrical, and photophysical properties of materials EA1–EA22.

	T_M_, °C	T_G_, °C	T_D_, °C	E_g_, eV	E_S_, eV	E_T_, eV	ΔE_ST_, eV	HOMO, eV	LUMO, eV	Φ_PL_ Film, %		Φ_PL_ Sol., %		R_D_, %
										N_2_	O_2_	N_2_	O_2_	
EA1	–	90	367	2.39	2.81	2.55	0.26	−5.55	−3.16	42.0	–	12.4	–	–
EA2	–	148	377	2.43	2.81	2.44	0.37	−5.58	−3.15	20.0	–	–	–	–
EA3	–	–	407	2.33	2.78	2.76	0.02	−5.51	−3.18	50.0	–	–	–	–
EA4	–	–	–	3.94	–	–	0.06	−5.83	−1.89	23.4	19.1	56.0	18.0	–
EA5	–	–	–	4.02	–	–	0.07	−5.99	−1.97	21.0	17.3	52.0	10.0	–
EA6	–	177	278	3.00	2.92	2.83	0.09	−5.45	−2.45	62.0	–	–	–	37.9
EA7	–	145	379	2.72	2.68	2.59	0.06	−5.37	−2.65	51.0	–	14.0	–	–
EA8	135	–	–	2.60	–	–	0.24	−5.20	−2.60	76.0	70	–	–	–
EA9	–	155	311	3.09	2.97	2.55	0.42	−5.66	−2.57	50.8	–	–	–	–
EA10	200	107	341	–	3.66	2.57	1.09	–	–	–	–	–	–	–
EA11	–	–	497	2.80	2.61	2.60	0.01	−5.35	−2.55	24.0	–	8.0	6.0	–
EA12	–	194	420	2.80	2.86	2.76	0.10	−5.90	−3.10	28.0	–	8.4	–	–
EA13	–	–	297	2.87	2.90	2.81	0.09	−5.06	−2.19	75.0	–	–	–	81.3
EA14	–	–	–	2.60	–	–	0.07	–	–	31.0	–	–	–	–
EA15	–	165	372	2.91	–	–	0.09	−5.11	−1.98	75.7	–	–	–	66.3
EA16	–	–	–	2.64	2.14	2.13	0.01	−5.70	−3.06	47.0	–	–	–	70.2
EA17	–	–	367	2.31	2.64	2.63	0.008	−5.24	−2.93	90.1	–	–	–	–
EA18	–	80	350	2.01	2.03	2.02	0.005	−5.24	−3.23	39.7	–	–	–	–
EA19	–	–	355	3.20	2.64	2.63	0.01	−4.93	−1.73	–	–	–	–	–
EA20	–	91	428	2.3	2.61	2.59	0.02	−5.50	−3.20	–	–	–	–	–
EA21	–	102	389	2.81	–	–	0.08	−5.12	−2.22	98.9	–	–	–	60.1
EA22	–	–	460	2.07	2.56	2.47	0.09	−5.02	−2.95	63.0	–	41.0	–	–

**Table 8:** Architectures of devices utilizing the emitters EA1–EA22.

Device	Device Architecture
DEA1	ITO/PEDOT:PSS (40 nm)/**EA1** (40 nm)/TPBi (30 nm)/Cs_2_CO_3_ (2 nm)/Al (100 nm)
DEA2	ITO/PEDOT:PSS (40 nm)/**EA2** (40 nm)/TPBi (30 nm)/Cs_2_CO_3_ (2 nm)/Al (100 nm)
DEA3	ITO/PEDOT:PSS (40 nm)/**EA3** (40 nm)/TPBi (30 nm)/Cs_2_CO_3_ (2 nm)/Al (100 nm)
DEA4	ITO/NPB (30 nm)/TAPC (20 nm)/mCP (10 nm)/DPEPO:7 wt% **EA4** (30 nm)/ TPBi (40 nm)/LiF (0.8 nm)/Al (100 nm)
DEA5	ITO/NPB (30 nm)/TAPC (20 nm)/mCP (10 nm)/DPEPO:7 wt% **EA5** (30 nm)/ TPBi (40 nm)/LiF (0.8 nm)/Al (100 nm)
DEA6	ITO/HAT-CN (10 nm)/TAPC (30 nm)/TCTA (10 nm)/DPEPO:10 wt% **EA6** (40 nm)/TmPyPB (40 nm)/LiF (1 nm)/Al (100 nm)
DEA7	ITO/PEDOT:PSS (25 nm)/**EA7** (25 nm)/TmPyPB (55 nm)/LiF (1 nm)/Al (150 nm)
DEA8	ITO (50 nm)/PEDOT:PSS (30 40 nm)/TCzl:5 wt% **EA8**/TPBi (50 nm)/Liq (1 nm)/Al (80 nm)
DEA9	ITO/TAPC (40 nm)/TCTA (20 nm)/**EA9** (40 nm)/TmPyPB (50 nm)/LiF (1 nm)/Al (100 nm)
DEA10	ITO (120 nm)/TAPC:20 wt%MoO_3_ (20 nm)/TAPC (20 nm)/TCTA (10 nm)/**EA10** (20 nm)/ CBP (2 nm)/TmPyPB (50 nm)/LiF (1.2 nm)/Al (120 nm)
DEA11	ITO/PEDOT:PSS (40 nm)/mCP:10 wt% **EA11** (40 nm)/TmPyPB (60 nm)/LiF (0.8 nm)/Al (100 nm)
D1EA12	ITO/NPB (30 nm)/TCTA (20 nm)/CzSi (10 nm)/**EA12** (20 nm)/DPEPO (10 nm)/TPBi (30 nm)/LiF (1 nm)/Al (100 nm)
D2EA12	ITO/NPB (30 nm)/TCTA (20 nm)/CzSi (10 nm)/DPEPO:20 wt% **EA12** (20 nm)/ DPEPO (10 nm)/TPBi (30 nm)/LiF (1 nm)/Al (100 nm)
D1EA13	ITO/MoO_3_ (1 nm)/TAPC (50 nm)/mCP (10 nm)/**EA13** (30 nm)/DPEPO (10 nm)/ TmPyPB (30 nm)/LiF (1 nm)/Al (100 nm)
D2EA13	ITO/MoO_3_ (1 nm)/TAPC (50 nm)/mCP (10 nm)/BCPO:20 wt% **EA13** (30 nm)/DPEPO (10 nm) /TmPyPB (30 nm)/LiF (1 nm)/Al (100 nm)
DEA14	ITO (100 nm)/α-NPD (40 nm)/mCBP (10 nm)/**EA14** (15 nm)/B3PyPB (55 nm)/Liq (1 nm)/Al (80 nm)
D1EA15	ITO/TAPC (30 nm)/mCP (10 nm)/**EA15** (20 nm)/DPEPO (10 nm)/TmPyPB (40 nm)/LiF/Al
D2EA15	ITO/TAPC (30 nm)/mCP (10 nm)/DPEPO:30 wt% **EA15** (20 nm)/DPEPO (10 nm)/TmPyPB (40 nm)/LiF/Al
DEA16	ITO/TAPC (40 nm)/TCTA (10 nm)/mCP (10 nm)/**EA16**/TmPyPb (40 nm)/LiF (1 nm)/Al (120 nm)
D1EA17	ITO/PEDOT:PSS (40 nm)/TAPC (20 nm)/**EA17** (20 nm)/TmPyPB (40 nm)/LiF (1 nm)/Al (200 nm)
D2EA17	ITO/PEDOT:PSS (40 nm)/TAPC (20 nm)/CBP:5 wt% **EA17** (20 nm)/TmPyPB (40 nm)/LiF (1 nm)/Al (200 nm)
D1EA18	ITO/PEDOT:PSS (40 nm)/TAPC (20 nm)/**EA18** (20 nm)/TmPyPB (40 nm)/LiF (1 nm)/Al (200 nm)
D2EA18	ITO/PEDOT:PSS (40 nm)/TAPC (20 nm)/CBP:5 wt% **EA18** (20 nm)/TmPyPB (40 nm)/LiF (1 nm)/Al (200 nm)
DEA19	ITO/PEDOT:PSS (30 nm)/mCP:CzAcSF:**EA19**(40:30:30) (40 nm) /DPEPO (10 nm)/TmPyPB (50 nm)/Liq (1 nm)/Al (100 nm)
D1EA20	ITO/PEDOT:PSS (40 nm)/CBP (20 nm)/CBP:10 wt% **EA20** (15 nm)/TPBi (40 nm)/Mg:Ag
D2EA20	ITO/PEDOT:PSS (40 nm)/CBP (20 nm)/**EA20** (4 nm)/2CzTPEPCz (15 nm)/TPBi (40 nm)/Mg:Ag
D1EA21	ITO/TAPC (30 nm)/mCP (10 nm)/**EA21** (20 nm)/DPEPO (10 nm)/TmPyPB (40 nm)/LiF/Al
D2EA21	ITO/TAPC (30 nm)/mCP (10 nm)/DPEPO:30 wt% **EA21** (20 nm)/DPEPO (10 nm)/TmPyPB (40 nm)/LiF/Al
DEA22	ITO/TAPC (30 nm)/TCTA (10 nm)/mCP:5 wt% **EA22** (20 nm)/TmPyPb (40 nm)/LiF (1 nm)/Al

**Table 9:** Characteristics of OLED devices using emitters EA1–EA22.

Device	Emitter	Colour	V_ON_, V	L_MAX_, cd/m^2^	CE, cd/A	PE, lm/W	EQE, %	CIE (x, y)
DEA1	EA1	Green	3.9	615	8.00	4.20	3.40	(0.27, 0.43)
DEA2	EA2	Blue	4.4	568	3.90	1.70	1.80	(0.21, 0.29)
DEA3	EA3	Green	3.8	1472	17.9	11.2	7.60	(0.26, 0.42)
DEA4	EA4	Blue	5.5	663	7.30	4.20	5.00	(0.18, 0.21)
DEA5	EA5	Blue	6.0	605	3.10	1.60	2.10	(0.19, 0.22)
DEA6	EA6	Blue	3.2	14,724	44.8	45.6	17.7	(0.17, 0.28)
DEA7	EA7	Green	2.5	>10,000	20.9	21.8	6.40	–
DEA8	EA8	Green	–	–	–	–	6.90	–
DEA9	EA9	Blue	4.0	10,754	23.3	19.3	9.50	(0.15, 0.15)
DEA10	EA10	Green	3.1	11,802	4.10	3.60	1.70	(0.23, 0.37)
DEA11	EA11	Green	4.7	17,353	24.8	–	8.90	(0.25, 0.48)
D1EA12	EA12	Green	3.8	2544	19.0	14.9	10.3	(0.26, 0.47)
D2EA12	EA12	Green	4.1	568	34.9	27.3	12.5	(0.23, 0.41)
D1EA13	EA13	Green	3.9	3006	18.2	14.1	6.59	(0.26, 0.47)
D2EA13	EA13	Green	3.2	13,280	44.4	45.0	15.2	(0.27, 0.50)
DEA14	EA14	Yellow	–	–	–	–	7.60	–
D1EA15	EA15	Green	3.2	6823	34.7	30.1	14.2	(0.20, 0,39)
D2EA15	EA15	Green	3.2	15,047	53.4	48.8	21.2	(0.21, 0.42)
DEA16	EA16	Green	3.0	–	32.7	34.3	12.9	(0.21, 0.42)
D1EA17	EA17	Green	2.5	21,243	53.7	52.7	17.3	(0.31, 0.54)
D2EA17	EA17	Green	3.2	11,977	49.6	48.6	17.7	(0.24, 0.49)
D1EA18	EA18	Yellow	2.5	11,949	8.90	7.99	3.35	(0.48, 0.50)
D2EA18	EA18	Green	2.7	31,115	64.6	75.1	20.6	(0.37, 0.55)
**DEA19**	**EA19**	**Blue**	**4.0**	**4235**	**47.7**	**29.9**	**20.6**	–
D1EA20	EA20	Yellow	4.4	32,590	73.1	38.2	26.7	–
D2EA20	EA20:2CzTPEPCz	White	6.1	12,310	45.9	18.0	20.8	(0.45, 0.44)
D1EA21	EA21	Green	3.2	26,836	56.4	43.5	18.7	(0.28, 0.53)
**D2EA21**	**EA21**	**Green**	**3.2**	**11,392**	**69.8**	**58.9**	**25.6**	**(0.24, 0.49)**
DEA22	EA22	Green	3.3	–	48.4	46.1	15.6	–

Characteristics of best performing blue and green devices are highlighted in bold.

**Table 10:** Thermal, electrochemical, and photophysical properties of materials EB1–EB27.

	T_M_, °C	T_G_, °C	T_Cr_, °C	T_D_, °C	E_g_, eV	E_S_, eV	E_T_, eV	ΔE_ST_, eV	HOMO, eV	LUMO, eV	Φ_PL_ Film, %		Φ_PL_ Sol., %	R_D_, %
											N_2_	O_2_		
EB1	–	107	–	384	2.95	2.62	2.47	0.15	−5.16	−2.21	13.1	–	2.20	87.8
EB2	–	91	–	312	3.21	–	3.02	–	−5.17	−2.03	20.0	–	3.00	–
EB3	–	–	–	–	3.10	2.89	3.10	0.21	−5.74	−2.64	55.0	–	21.0	–
EB4	370	–	–	480	3.30	2.83	2.69	0.14	−5.77	−2.25	24.4	–	15.3	–
EB5	–	–	–	–	3.02	3.02	2.88	0.14	−5.65	−2.63	73.0	–	38.0	–
EB6	–	–	–	–	2.60	–	–	0.29	−5.10	−2.50	73.0	37.0	–	–
EB7	–	159	–	398	2.79	–	–	–	−5.58	−2.79	–	–	–	–
EB8	–	–	–	–	–	–	–	0.39	–	–	53.0	–	–	7.5
EB9	342	–	269	389	3.66	2.70	2.66	0.04	−4.78	−219	16.0	–	6.00	–
EB10	–	–	–	410	–	2.76	2.69	0.07	–	–	90.0	–	–	–
EB11	–	–	–	433	2.82	–	–	0.03	−5.16	−2.34	89.1	–	–	–
EB12	–	–	–	270	2.86	2.81	2.68	0.13	−5.26	−2.40	31.5	–	–	32.7
EB13	118	–	62	218	3.05	–	2.65	–	−4.74	−2.19	15.0	–	12.0	–
EB14	–	–	–	–	2.52	2.61	2.58	0.03	−5.44	−2.92	70.0	–	44.0	–
EB15	–	–	–	–	2.61	2.62	2.52	0.10	−5.64	−3.03	71.0	–	36.0	–
EB16	–	–	–	–	2.49	2.59	2.53	0.06	−5.62	−3.13	36.0	–	10.0	–
EB17	–	90	–	436	2.84	2.59	2.38	0.21	−5.31	−2.47	75.0	–	46.0	–
EB18	–	92	–	416	2.70	2.37	2.32	0.05	−5.32	−2.62	39.0	–	2.00	–
EB19	–	99	–	440	2.90	2.66	2.47	0.19	−5.51	−2.60	16.3	–	–	–
EB20	–	–	–	–	–	2.84	2.69	0.15	–	–	7.60	–	–	–
EB21	–	–	–	–	–	2.84	2.69	0.15	–	–	8.50	–	–	–
EB22	300	–	–	495	2.94	2.71	2.64	0.07	−5.49	−2.55	70.0	–	–	–
EB23	–	–	–	445	3.37	2.62	2.61	0.01	−5.42	−2.05	91.0	–	–	55.6
EB24	–	–	–	477	3.32	2.64	2.63	0.01	−5.42	−2.10	94.0	–	–	54.5
EB25	–	–	–	500	3.32	2.64	2.63	0.01	−5.43	−2.11	85.0	–	–	42.8
EB26	–	>95	–	>400	2.47	2.55	2.47	0.13	−5.30	−2.83	22.0	–	0.80	–
EB27	–	>95	–	>400	2.59	2.58	2.48	0.13	−5.33	−2.77	24.2	–	1.90	–

**Table 11:** Architectures of devices utilizing the D–A–D emitters EB1–EB27.

Device with EB Emitter	Device Architecture
DEB3	ITO/α-NPD (35 nm)/mCP (5 nm)/DPEPO:6 wt% **EB3** (20 nm)/ DPEPO (10 nm)/TPBi (30 nm)/LiF (0.8 nm)/Al (80 nm)
D1EB4	ITO/NPB (35 nm)/mCP (5 nm)/**EB4** (30 nm)/B3PYMPM (30 nm)/LiF (0.5 nm)/Al (150 nm)
D2EB4	ITO/NPB (35 nm)/mCP (5 nm)/**EB4**:0.5%wt Ir(ppy)_2_(acac)/**EB4**:0.8 wt% Ir(bt)_2_(dipba) (30 nm)/ B3PYMPM (30 nm)/LiF (0.5 nm)/Al (150 nm)
DEB5	ITO/α-NPD (35 nm)/mCP (5 nm)/DPEPO:6 wt% **EB5** (20 nm)/DPEPO (10 nm)/ TPBi (30 nm)/LiF (0.8 nm)/Al (80 nm)
DEB6	ITO(50 nm)/PEDOT:PSS (30 40 nm)/TCzl:15 wt% **EB6**/TPBi (70 nm)/Liq (1 nm)/Al (80 nm)
DEB8	ITO/TAPC (25 nm)/CBP:3 wt% **EB8** (35 nm)/TmPyPB (55 nm)/LiF (1 nm)/Al
DEB9	ITO/TAPC (50 nm)/mCP (10 nm)/**EB9** (30 nm)/Bphen (50 nm)/LiF (1 nm)/Al (100 nm)
DEB10	ITO/MoO_3_ (1 nm)/mCP (40 nm)/**EB10** (30 nm)/TBPi (50 nm)/LiF (1 nm)/Al
DEB11	ITO/PEDOT:PSS (40 nm)/mCPCN:25 wt% **EB11** (45 nm)/ DPEPO (10 nm)/TmPyPB (40 nm)/Liq (1.2 nm)/Al (120 nm)
DEB12	ITO/HAT-CN (5 nm)/TAPC (30 nm)/TCTA (5 nm)/mCP (5 nm)/PPF:20% **EB12**(30 nm)/ PPF (10 nm)/Bphen (30 nm)/Liq (1 nm)/Al (100 nm)
DEB14	ITO/α-NPD (40 nm)/mCP:6 wt% **EB14** (20 nm)/TPBi (40 nm)/LiF (0.8 nm)/Al (80 nm)
DEB15	ITO/α-NPD (40 nm)/**EB15** (20 nm)/TPBi (40 nm)/LiF (0.8 nm)/Al (80 nm)
DEB16	ITO/α-NPD (40 nm)/mCBP:6 wt% **EB16** (20 nm)/TPBi (40 nm)/LiF (0.8 nm)/Al (80 nm)
DEB16EB5	ITO/α-NPD (35 nm)/mCBP:18 wt% **EB16** (4 nm)/PPF:6 wt%**EB5** (14 nm)/PPF (40 nm)/LiF (0.8 nm)/Al (80 nm)
DEB17	ITO/PEDOT:PSS (40 nm)/**EB17** (35–40 nm)/TmPyPB (60 nm)/LiF (0.8 nm)/Al (120 nm)
DEB18	ITO/PEDOT:PSS (40 nm)/**EB18** (35–40 nm)/TmPyPB (60 nm)/LiF (0.8 nm)/Al (120 nm)
DEB19	ITO/Mo_2_O_3_ (4 nm)/mCP (30 nm)/mCP:15 wt% **EB19** (30 nm)/TmTyPB (60 nm)/LiF (1.5 nm)/Al (100 nm)
DEB20	ITO/α-NPD (40 nm)/mCBP:10 wt% **EB20** (20 nm)/TPBi (40 nm)/LiF (0.6 nm)/Al (100 nm)
DEB21	ITO/α-NPD (40 nm)/mCBP:10 wt% **EB21** (20 nm)/TPBi (40 nm)/LiF (0.6 nm)/Al (100 nm)
DEB22	ITO (70 nm)/4 wt%ReO_3_:mCP (45 nm)/mCP (15 nm)/mCP:TSPO1:16 wt%**EB22** (15 nm)/ TSPO1 (15 nm)/4 wt%Rb_2_CO_3_:TSPO1 (50 nm)/Al (100 nm)
D1EB23	ITO/HAT-CN (5 nm)/NPB (30 nm)/mCP (5 nm)/**EB23** (20 nm)/PPF (5 nm)/TPBi (50 nm)/LiF (1 nm)/Al (120 nm)
D2EB23	ITO/HAT-CN (5 nm)/NPB (30 nm)/mCP (5 nm)/PPF:30 wt% **EB23** (20 nm)/ PPF (5 nm)/TPBi (50 nm)/LiF (1 nm)/Al (120 nm)
D1EB24	ITO/HAT-CN (5 nm)/NPB (30 nm)/mCP (5 nm)/**EB24** (20 nm)/PPF (5 nm)/TPBi (50 nm)/LiF (1 nm)/Al (120 nm)
D2EB24	ITO/HAT-CN (5 nm)/NPB (30 nm)/mCP (5 nm)/PPF:30 wt% **EB24** (20 nm)/PPF (5 nm)/ TPBi (50 nm)/LiF (1 nm)/Al (120 nm)
D1EB25	ITO/HAT-CN (5 nm)/NPB (30 nm)/mCP (5 nm)/**EB25** (20 nm)/PPF (5 nm)/TPBi (50 nm)/LiF (1 nm)/Al (120 nm)
D2EB25	ITO/HAT-CN (5 nm)/NPB (30 nm)/mCP (5 nm)/PPF:30 wt% **EB25** (20 nm)/PPF (5 nm)/ TPBi (50 nm)/LiF (1 nm)/Al (120 nm)
D1EB26	ITO/PEDOT:PSS (25 nm)/**EB26** (25 nm)/TmPyPB (55 nm)/LiF (1 nm)/Al (150 nm)
D2EB26	ITO/PEDOT:PSS (25 nm)/G3-tCbz:30 wt% **EB26** (25 nm)/TmPyPB (55 nm)/LiF (1 nm)/Al (150 nm)
D1EB27	ITO/PEDOT:PSS (25 nm)/**EB27** (25 nm)/TmPyPB (55 nm)/LiF (1 nm)/Al (150 nm)
D1EB27	ITO/PEDOT:PSS (25 nm)/G3-tCbz:30 wt% **EB27** (25 nm)/TmPyPB (55 nm)/LiF (1 nm)/Al (150 nm)

**Table 12:** Characteristics of OLED devices using emitters EB1–EB27.

Device	Emitter	Colour	V_ON_, V	L_MAX_, cd/m^2^	CE, cd/A	PE, lm/W	EQE, %	CIE (x, y)
DEB3	EB3	Blue	4.3	510	9.30	–	8.10	(0.16, 0.14)
D1EB4	EB4	Blue	3.2	5516	–	6.90	4.00	(0.15, 0.26)
D2EB4	EB4/Ir(ppy)_2_(acac)/Ir(bt)_2_(dipba)	White	2.7	25,540	48.6	–	25.6	(0.41, 0.46)
**DEB5**	**EB5**	**Blue**	**4.4**	**3900**	**25.5**	–	**14.3**	**(0.17, 0.27)**
DEB6	EB6	Green	–	–	–	–	10.4	–
DEB8	EB8	Blue	–	–	–	–	8.90	(0.14, 0.16)
DEB9	EB9	Green	7.5	114	11.8	9.30	1.93	(0.37, 0.57)
DEB10	EB10	Green	2.6	45,300		59.0	18.0	(0.26, 0.55)
DEB11	EB11	Green	4.4	7173	69.3	–	22.5	(0.29, 0.54)
DEB12	EB12	Green	5.3	3524	11.0	6.90	3.90	(0.28, 0.51)
DEB14	EB14	Green	3.2	86,100	35.9	–	10.7	(0.37, 0.58)
DEB15	EB15	Red	2.8	50,820	11.1	–	4.20	(0.58, 0.36)
DEB16	EB16	Yellow	3.6	57,120	20.1	–	6.90	(0.49, 0.51)
DEB16/EB5	EB16/EB5	White	5.0	9800	16.4	–	6.70	(0.32, 0,39)
DEB17	EB17	Blue	3.9	10,005	4.80	2.70	2.40	(0.15, 0.28)
DEB18	EB18	Green	3.8	7354	10.8	4.70	2.70	(0.33, 0.51)
DEB19	EB19	Blue	3.9	1387	2.74	2.20	1.69	(0.16, 0.21)
DEB20	EB20	Green	4.3	17,007	64.0	–	30.8	(0.31, 0.53)
DEB21	EB21	Green	5.4	16,883	73.5	–	18.8	(0.33, 0.48)
DEB22	EB22	Blue	3.6	2021	–	–	11.4	(0.17, 0.31)
D1EB23	EB23	Green	3.2	31,713	53.9	48.9	18.6	(0.24, 0.53)
D2EB23	EB23	Green	3.0	48,712	90.9	91.2	30.3	(0.25, 0.54)
D1EB24	EB24	Green	3.6	30,283	46.8	37.5	17.1	(0.22, 0.46)
**D2EB24**	**EB24**	**Green**	**3.2**	**48,515**	**87.5**	**85.9**	**32.2**	**(0.22, 0.49)**
D1EB25	EB25	Green	3.8	25,616	49.1	35.7	18.1	(0.22, 0.50)
D2EB25	EB25	Green	3.2	48,153	79.8	75.9	28.4	(0.23, 0.50)
D1EB25	EB25	Yellow	2.6	>10,000	17.8	20.0	5.90	(0.41, 0.54)
D2EB25	EB25	Yellow-green	2.8	>10,000	48.1	47.8	15.9	(0.37, 0.53)
D1EB26	EB26	Yellow-green	2.7	>10,000	18.9	19.2	6.00	(0.38, 0.55)
D2EB26	EB26	Green	2.8	>10,000	46.4	47.2	15.4	(0.34, 0.53)

Characteristics of best performing blue and green devices are highlighted in bold.

**Table 13:** Thermal, electrochemical, and photophysical properties of materials EC1–EC11.

	T_M_, °C	T_G_, °C	T_D_, °C	E_g_, eV	E_S_, eV	E_T_, eV	ΔE_ST_, eV	HOMO, eV	LUMO, eV	Φ_PL_ Film, %	Φ_PL_ Sol., %	R_D_, %
EC1	–	–	433	2.30	2.75	2.71	0.04	−5.36	−3.06	57	–	78.9
EC2	–	72	309	2.76	2.82	2.72	0.10	−5.37	−2.61	–	–	–
EC3	–	–	417	2.53	2.67	2.66	0.013	−5.35	−2.82	90	28	53.3
EC4	–	–	451	2.58	2.71	2.66	0.05	−5.36	−2.78	86	25	33.0
EC5	–	–	404	2.36	2.81	2.71	0.10	−5.35	−2.99	46.7	1.2	76.4
EC6	–	–	414	2.30	2.69	2.65	0.04	−5.36	−3.06	66.8	3.4	89.7
EC7	–	–	416	2.25	2.59	2.56	0.03	−5.34	−3.09	53.3	0.5	83.3
EC8	–	–	391	2.34	2.58	2.52	0.06	−5.26	−2.92	33.2	0.6	75.3
EC9	–	–	400	2.26	2.57	2.54	0.03	−5.25	−2.99	48.4	0.9	88.2
EC10	–	–	403	2.19	2.41	2.38	0.03	−5.23	−3.04	35.3	0.8	82.7
EC11	190	104	416	2.88	2.85	2.79	0.06	−5.92	−3.04	76	17	–

**Table 14:** Architectures of the devices utilizing emitters EC1–EC11.

Device	Device Architecture
DEC1	ITO/PEDOT:PSS (40 nm)/**EC1** (40 nm)/TPBi (30 nm)/Cs_2_CO_3_ (2 nm)/Al (100 nm)
DEC2	ITO/HAT-CN (20 nm)/TAPC (30 nm)/CBP:20 wt% **EC2** (25 nm)/TmPyPb (40 nm)/LiF (1 nm)/Al (150 nm)
D1EC3	ITO/HAT-CN (5 nm)/NPB (30 nm)/mCP (5 nm)/**EC3** (20 nm)/PPF (5 nm)/TPBi (50 nm)/LiF (1 nm)/Al (120 nm)
D2EC3	ITO/HAT-CN (5 nm)/NPB (30 nm)/mCP (5 nm)/PPF:20 wt% **EC3** (20 nm)/ PPF (5 nm)/TPBi (50 nm)/LiF (1 nm)/Al (120 nm)
D1EC4	ITO/HAT-CN (5 nm)/NPB (30 nm)/mCP (5 nm)/**EC4** (20 nm)/PPF (5 nm)/TPBi (50 nm)/LiF (1 nm)/Al (120 nm)
D2EC4	ITO/HAT-CN (5 nm)/NPB (30 nm)/mCP (5 nm)/PPF:20 wt% **EC4** (20 nm)/ PPF (5 nm)/TPBi (50 nm)/LiF (1 nm)/Al (120 nm)
DEC5	ITO/PEDOT:PSS (40 nm)/**EC5** (40 nm)/TPBi (30 nm)/Cs_2_CO_3_ (2 nm)/Al (100 nm)
DEC6	ITO/PEDOT:PSS (40 nm)/**EC6** (40 nm)/TPBi (30 nm)/Cs_2_CO_3_ (2 nm)/Al (100 nm)
DEC7	ITO/PEDOT:PSS (40 nm)/**EC7** (40 nm)/TPBi (30 nm)/Cs_2_CO_3_ (2 nm)/Al (100 nm)
DEC8	ITO/PEDOT:PSS (40 nm)/**EC8** (40 nm)/TPBi (30 nm)/Cs_2_CO_3_ (2 nm)/Al (100 nm)
DEC9	ITO/PEDOT:PSS (40 nm)/**EC9** (40 nm)/TPBi (30 nm)/Cs_2_CO_3_ (2 nm)/Al (100 nm)
DEC10	ITO/PEDOT:PSS (40 nm)/**EC10** (40 nm)/TPBi (30 nm)/Cs_2_CO_3_ (2 nm)/Al (100 nm)
DEC11	ITO/MoO_3_ (1.2 nm)/NPB (44 nm)/TCTA (4 nm)/mCP (4 nm)/mCP:7%wt **EC11** (24 nm)/ TSPO1 (4 nm)/TPBi (40 nm)/LiF (1.2 nm)/Al (400 nm)

**Table 15:** Characteristics of OLED devices using emitters EC1–EC11.

Device	Emitter	Colour	V_ON_, V	L_MAX_, cd/m^2^	CE, cd/A	PE, lm/W	EQE, %	CIE (x, y)
DEC1	EC1	Green	3.8	1738	35.5	22.3	13.3	(0.29, 0.48)
DEC2	EC2	Green	3.6	116,000	61.8	40.4	19.7	(0.26, 0.56)
D1EC3	EC3	Green	3.2	78,540	76.9	71.0	29.0	(0.21, 0.47)
D2EC3	EC3	Green	3.2	71,150	82.9	70.1	33.3	(0.20, 0.42)
D1EC4	EC4	Green	3.0	54,450	53.2	51.5	21.6	(0.20, 0.42)
**D2EC4**	**EC4**	**Green**	**3.2**	**42,550**	**77.2**	**65.0**	**32.9**	**(0.19, 0.38)**
DEC5	EC5	Green	4.5	2599	14.3	6.40	6.70	(0.28, 0.47)
DEC6	EC6	Green	4.2	6726	35.4	15.9	11.4	(0.34, 0.51)
DEC7	EC7	Green	4.3	3832	23.8	10.7	9.10	(0.39, 0.56)
DEC8	EC8	Yellow	4.8	3658	12.4	4.30	4.80	(0.41, 0.55)
DEC9	EC9	Orange	4.6	8085	21.6	6.80	9.40	(0.47, 0.51)
DEC10	EC10	Orange	4.7	6551	14.5	4.20	6.90	(0.53, 0.46)
DEC11	EC11	Green	4.2	15,600	57.8	38.8	22.2	(0.18, 0.41)

Characteristics of best performing device are highlighted in bold.

**Table 16:** Thermal, electrochemical, and photophysical properties of materials ED1–ED10.

	T_M_, °C	T_G_, °C	T_D_, °C	E_g_, eV	E_S_, eV	E_T_, eV	ΔE_ST_, eV	HOMO, eV	LUMO, eV	Φ_PL_ Film, %	Φ_PL_ Sol., %	R_D_, %
ED1	–	218	–	2.73	2.80	2.72	0.08	−5.82	−3.09	74.0	49.0	45.9
ED2	–	–	–	2.74	2.82	2.73	0.09	−5.72	−2.98	34.0	48.0	17.6
ED3	–	–	–	2.67	2.65	2.54	0.11	−5.43	−2.76	17.0	74.0	5.90
ED4	–	–	–	2.92	2.83	2.69	0.14	−5.81	−2.89	41.0	32.0	17.1
ED5	–	–	–	2.96	2.84	2.72	0.12	−5.87	−2.91	33.4	23.6	40.1
ED6	–	–	–	3.06	2.84	2.74	0.10	−5.73	−2.67	21.1	14.7	64.5
ED7	313	283	471	2.58	2.69	2.58	0.11	−5.12	−2.54	77.0	–	–
ED8	367	289	507	2.56	2.84	2.69	0.15	−5.25	−2.69	75.0	–	–
ED9	–	–	493	3.01	–	–	0.13	−5.04	−2.03	21.7	–	18.4
ED10	–	–	522	3.12	–	–	0.11	−5.05	−2.17	36.5	–	9.00

**Table 17:** Architectures of devices utilizing emitters ED1–ED10.

Device	Device Architecture
D1ED1	ITO/PEDOT:PSS (40 nm)/**ED1** (40 nm)/TPBI (30 nm)/Cs_2_CO_3_ (2 nm)/Al (120 nm)
D2ED1	ITO/PEDOT:PSS (50 nm)/PVK (20 nm)/**ED1** (25 nm)/SPPO13 (40 nm)/LiF (0.5 nm)/Al (80 nm)
DED2	ITO/PEDOT:PSS (50 nm)/PVK (20 nm)/**ED2** (25 nm)/SPPO13 (40 nm)/LiF (0.5 nm)/Al (80 nm)
DED3	ITO/PEDOT:PSS (50 nm)/PVK (20 nm)/**ED3** (25 nm)/SPPO13 (40 nm)/LiF (0.5 nm)/Al (80 nm)
DED4	ITO/PEDOT:PSS (50 nm)/PVK (20 nm)/**ED4** (25 nm)/SPPO13 (40 nm)/LiF (0.5 nm)/Al (80 nm)
DED5	ITO/PEDOT:PSS (70 nm)/PVK (20 nm)/**ED5** (30 nm)/TPBi (40 nm)/Ca (10 nm)/Al (80 nm)
DED6	ITO/PEDOT:PSS (70 nm)/PVK (20 nm)/**ED6** (30 nm)/TPBi (40 nm)/Ca (10 nm)/Al (80 nm)
DED7	ITO/PEDOT:PSS (30 nm)/**ED7** (70 nm)/TPBi (40 nm)/Liq (2 nm)/Al
DED8	ITO/PEDOT:PSS (30 nm)/**ED8** (70 nm)/TPBi (40 nm)/Liq (2 nm)/Al
D1ED9	ITO/TAPC (30 nm)/mCP (10 nm)/**ED9** (20 nm)/DPEPO (30 nm)/DPEPO (10 nm) /TmPyPB (40 nm)/LiF/Al
D2ED9	ITO/TAPC (30 nm)/mCP (10 nm)/DPEPO:30 wt% **ED9** (20 nm)/DPEPO (30 nm)/DPEPO (10 nm) /TmPyPB (40 nm)/LiF/Al
D1ED10	ITO/TAPC (30 nm)/mCP (10 nm)/**ED10** (20 nm)/DPEPO (30 nm)/DPEPO (10 nm)/ TmPyPB (40 nm)/LiF/Al
D2ED10	ITO/TAPC (30 nm)/mCP (10 nm)/DPEPO:30 wt% **ED10** (20 nm)/DPEPO (30 nm)/DPEPO (10 nm)/ TmPyPB (40 nm)/LiF/Al

**Table 18:** Characteristics of OLED devices using emitters ED1–ED10.

Device	Emitter	Colour	V_ON_, V	L_MAX_, cd/m^2^	CE, cd/A	PE, lm/W	EQE, %	CIE (x, y)
D1ED1	ED1	Green	4.5	4200	9.20	–	4.30	(0.26, 0.46)
**D2ED1**	**ED1**	**Green**	**2.7**	**4639**	**46.6**	**40.7**	**17.0**	**(0.27, 0.52)**
DED2	ED2	Green	2.6	661	23.5	25.0	9.00	(0.28, 0.48)
DED3	ED3	Yellow	2.5	1017	17.7	19.0	6.40	(0.44, 0.51)
DED4	ED4	Green	2.7	965	22.7	20.0	8.80	(0.30, 0.48)
DED5	ED5	Green	3.4	–	14.0	11.5	5.70	(0.26, 0.48)
DED6	ED6	Green	3.7	–	8.70	6.60	3.60	(0.28, 0.43)
DED7	ED7	Green-yellow	4.8	>10,000	–	–	12.0	(0.38, 0.56)
DED8	ED8	Green	7.7	2512	–	–	5.20	(0.32, 0.51)
D1ED9	ED9	Blue	4.4	1290	9.70	6.10	4.20	(0.19, 0.36)
**D2ED9**	**ED9**	**Blue**	**3.0**	**2189**	**28.5**	**24.9**	**13.4**	**(0.18, 0.33)**
D1ED10	ED10	Green	4.4	5949	24.0	15.6	8.50	(0.26, 0.50)
D2ED10	ED10	Green-blue	3.0	3867	44.4	42.8	18.9	(0.20, 0.38)

Characteristics of best performing green and blue devices are highlighted in bold.

## Data Availability

The data presented in this study are available upon request from the corresponding authors.
